# Membrane Bioprocesses for Pharmaceutical Micropollutant Removal from Waters

**DOI:** 10.3390/membranes4040692

**Published:** 2014-10-06

**Authors:** Matthias de Cazes, Ricardo Abejón, Marie-Pierre Belleville, José Sanchez-Marcano

**Affiliations:** Institut Européen des Membranes (IEM), ENSCM, UM2, CNRS, Université de Montpellier 2, CC 047, Place Eugène Bataillon 34095, France; E-Mails: matthias.de-cazes@iemm.univ-montp2.fr (M.C.); ricardo.abejon@iemm.univ-montp2.fr (R.A.); marie-pierre.belleville@iemm.univ-montp2.fr (M.-P.B.)

**Keywords:** membrane bioprocesses, pharmaceutical micropollutants, wastewaters, membrane bioreactors, enzymatic membrane reactors

## Abstract

The purpose of this review work is to give an overview of the research reported on bioprocesses for the treatment of domestic or industrial wastewaters (WW) containing pharmaceuticals. Conventional WW treatment technologies are not efficient enough to completely remove all pharmaceuticals from water. Indeed, these compounds are becoming an actual public health problem, because they are more and more present in underground and even in potable waters. Different types of bioprocesses are described in this work: from classical activated sludge systems, which allow the depletion of pharmaceuticals by bio-degradation and adsorption, to enzymatic reactions, which are more focused on the treatment of WW containing a relatively high content of pharmaceuticals and less organic carbon pollution than classical WW. Different aspects concerning the advantages of membrane bioreactors for pharmaceuticals removal are discussed, as well as the more recent studies on enzymatic membrane reactors to the depletion of these recalcitrant compounds.

## 1. Introduction

Pharmaceutical compounds have been continuously released in the environment since their first applications for human or veterinarian purposes at the end of the 19th century. They are used world-wide, and improved living conditions, as well as the growing demography have led to their constantly increasing discharges around the world. Pharmaceuticals represent more than 4000 different molecules with a production of several 100,000 tons per year. Although conventional wastewater treatment technologies are efficient for a large range of compounds, some persistent organic micropollutants are very resistant, and traces can be found at the output of the wastewaters plants.

Data regarding pharmaceuticals consumption around the world are difficult to compare, because they vary each year and depend on the therapeutic doses and the prescription rate, which are specific to each country. A few studies managed to measure the trend of antibiotics uptake, and the most commonly used are acetaminophen, clarithromycin, ibuprofen, carbamazepine, ciprofloxacin, erythromycin, sulfamethoxazole and tetracycline [1,2,3,4,5].

Water Framework Directive 2000/60/CE from 23 October 2000 is a management plan that aims at achieving a good water quality in 2015 by progressively reducing emissions of priority substances and eliminating dangerous compound discharges in 2021 with wastewater treatments improvement. The preservation of the aquatic environment can require the modification of emissions limits for specific effluents containing micropollutants. Thus, a good ecological and chemical state of surface and ground water will be expected.

Pharmaceutical pollutants found in waters come from several contamination sources, such as urban and industrial wastewaters, agriculture, aquaculture or soil contamination in animal husbandry for therapy or growth promoter purposes [6,7,8]. After being consumed by humans or animals, some of these compounds are metabolized, while others remain un-metabolized and are ultimately eliminated from the body. Depending on the compounds, their uptake by metabolism can reach from 10% to 90%. A mix of metabolites and medicines can be found in municipal wastewaters and sludge. Effluents discharged from drug manufacturing plants make the most significant contribution to the total pharmaceutical concentration in water [9,10]. Sewage sludge can be sometimes used as fertilizer, and its pollutants reach soils through irrigation systems, spreading them through the ground and across agriculture [11,12]. Recent studies showed that antibiotics have very high half-lives when they reach agricultural soils: 60 to 495 days for carbamazepine, 55 to 578 days for tetracycline and even 120 to 2310 days for ciprofloxacin [13].

However, as far as these being relatively diluted in wastewaters, only the development of sensible-enough analytical methods has opened up the possibility to identify and monitor them in water effluents. In the past, they have therefore not been considered as priority pollutants to target. Some studies have noticed the presence of pharmaceutical compounds, as well as their transformation products at the exit of wastewater treatment plants in surface water, in groundwater, adsorbed on sediments and even in drinking water [7,14,15].

Conventional biological treatments are able to deplete, to some extent, several pharmaceuticals or, even completely, some of these compounds, as will be explained later. However, the whole removal of pharmaceutical compounds from water is not possible with conventional wastewater treatment technologies. These compounds are indeed relatively resistant to widely used decontamination techniques and discharged in treated wastewaters.

**Figure 1:**
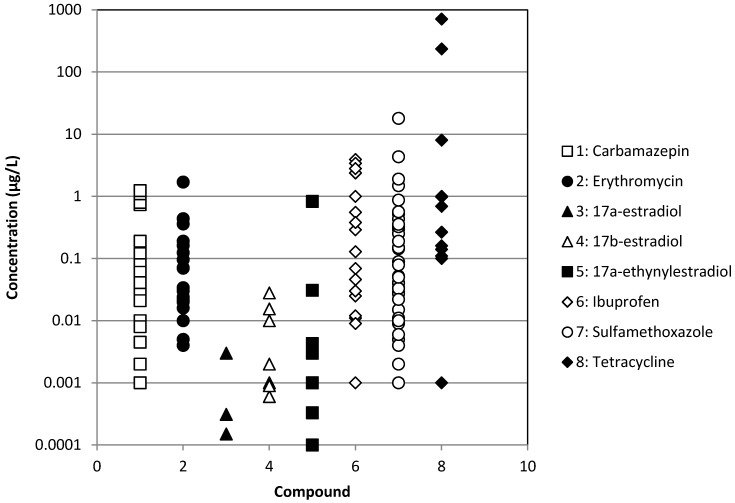
Occurrence of some pharmaceuticals in treated municipal wastewaters.

**Figure 2:**
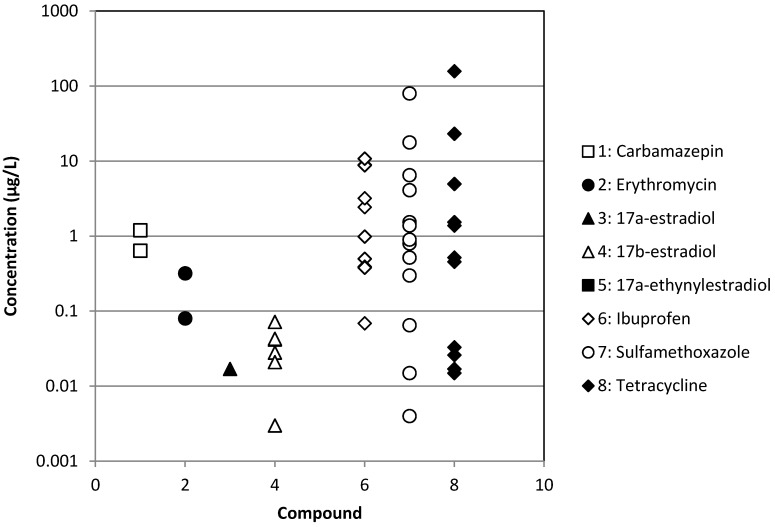
Occurrence of some pharmaceuticals in treated hospital wastewaters.

**Figure 3:**
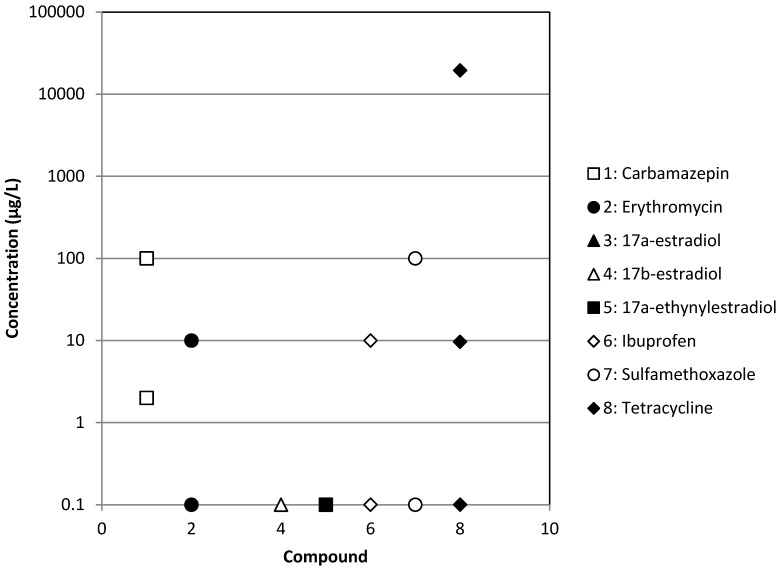
Occurrence of some pharmaceuticals in industrial wastewaters.

**Figure 4:**
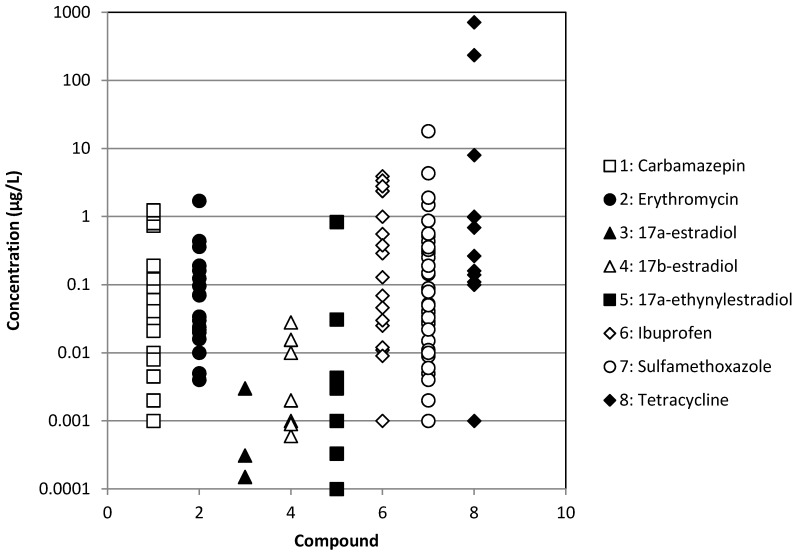
Occurrence of some pharmaceuticals in raw surface waters.

The occurrence of different types of pharmaceuticals from antibiotics to antiepileptic drugs or hormones in different types of treated or raw wastewaters has been reported in Figure 1, Figure 2, Figure 3 and Figure 4. These Figures were built with the references given in Table 1 and concern effluents of wastewater treatment plants (WWTP) of three origins: municipal, hospital and industrial and raw surface waters (rivers, lakes, ponds, *etc*.). We can notice that the concentration is very variable, but always ranges between 10^−4^ and 10^2^ µg·L^−1^ for treated municipal and hospital wastewaters; even if in the case of municipal wastewaters, the volumes and dilution have to be much higher than in the case of hospitals. As expected, industrial wastewaters that come from pharmaceutical production present the highest pollutant content (between 10^−1^ and 10^4^ µg·L^−1^), whereas surprisingly, raw surface waters from rivers, lakes and ponds present a relatively high content (between 10^−4^ and 10^3^ µg·L^−1^) of some pollutants, like tetracycline, a well-known antibiotic. Moreover, the results reported for tetracycline are relatively high among the different pollutants reported, although this antibiotic is well known for its self-degradation initiated by solar radiation [16]. This result is a good indication of the very extensive use of this pharmaceutical.

The risks of the long-term toxicity of pharmaceuticals are not well-known and may have a deep influence on the evolution of aquatic and terrestrial ecosystems and their fauna and flora. Eco-toxicity studies have demonstrated that pharmaceutical pollutants could affect the growth, reproduction and behavior of birds, fishes, invertebrates, plants and bacteria, even at levels as low as a few ng·L^−1^ [17,18,19,20,21]. Human health is threatened by the presence of trace concentrations in soils, which are directly connected to food and drinking water. Even if absorbed quantities from water or food are below therapeutic concentrations and no acute toxicity is observable, the long-term effects are still unpredictable and undocumented [5,22]. The emergence of drug-resistant pathogens is another concern for human health. Infections with antibiotic-resistant bacteria form a major and increasing cause of mortality in hospitals [23]. Some recently published studies report that the occurrence of very low concentrations of antibiotics in wastewaters may be at the origin of antibiotic resistance in the whole environment [24,25].

The objective of this review work is to present the latest developments and research reported on membrane bioreactors (microbial or enzymatic) for the depletion of recalcitrant pharmaceutical compounds in wastewaters.

**Table 1:** Literature review of the content of pharmaceuticals in effluents from wastewater treatment plants from municipal, hospital and industrial waters and raw surface waters. The number of the reference is in brackets. WWTP, wastewater treatment plants.

Chemical	WWTP Effluents			Surface waters
	Municipal	Hospital	Industrial	
Ibuprofen	[3,8,12,15,26,27,28,29,30,31,32,33,34,35,36]	[33,34,36]	[37]	[3,6,8,28,30,38,39,40,41,42]
Erythromycin	[3,7,15,29,32,34,40,43,44,45,46]	[34]	[37]	[3,6,7,39,46,47,48,49]
Sulfamethoxazole	[2,3,7,8,15,26,27,28,29,30,32,33,34,40,43,46,50,51,52,53,54,55,56]	[33,34,50,55]	[37]	[2,3,6,7,8,28,30,38,39,40,41,46,47,48,49,50,57,58]
Tetracycline	[3,7,15,29,44,51,52,53,56,59,60,61]	[33,34,59,61]	[9,37,59]	[3,6,7,9,38,39,48,59,61]
Carbamazepine	[2,3,8,15,26,27,28,29,32,34,40,62]	[34]	[37]	[2,3,8,28,38,39,40,41,42,62]
17α-estradiol	[3,8,29,36,63]	[36]		[8,63]
17α-ethinylestradiol	[3,8,29,32,63,64]		[37]	[8,39,63,64]
17β-estradiol	[3,8,29,32,45,63,64,65]	[33]	[37]	[8,63,64,65]

## 2. Biological Treatments

As explained above, classical biological treatments are not able to deplete completely all of the pharmaceuticals present in wastewaters. However, some microorganisms are able to metabolize these molecules and even to totally degrade some of them. Current wastewater treatment processes always involve biological technologies; among the different processes used, the activated sludge (AS) system is the most common one. It is based on aeration and agitation of wastewater, which contains a very large spectra biomass population; some strain consortiums are able to degrade classical macro pollutants (C, N, P), whereas other consortiums are able to adapt to particular pollutants, like chemicals, allowing their degradation. As reported in recent reviews [63,66,67], some bacteria are able to assimilate and transform pharmaceutical micropollutants, like endocrine disrupting compounds (EDCs) or antibiotics, as long as the conditions are favorable for biomass growth. In fact, pharmaceutical micropollutants are highly biologically active molecules, and they could have a negative impact on the metabolism of microorganisms. Onesios and Bouwer, who studied the biological removal of pharmaceuticals compounds, as well as personal care products (PPCPs), showed that a mixture of PPCPs can suppress biofilm growth [68]. Biodegradation of PPCPs was also studied in anaerobic conditions [69,70,71]. The removal rates obtained depend on the targeted compounds. Hormones can be degraded by anaerobic bacteria, but only to some extent. Indeed, although endocrine disruptors, such as 17β-estradiol, could be converted to estrone or 17α-estradiol, the decrease of the estrogenicity of the water remains suggesting that those compounds would accumulate in anoxic environments [71]. Nevertheless, Carballa *et al*. reported excellent removal efficiencies (>85%) in anaerobic conditions for natural estrogens, musks and some pharmaceuticals, like antibiotics and naproxen; however, carbamazepine was not depleted in such conditions [70]. It was also reported that the use of an activated sludge system for nitrification and denitrification can be very useful to degrade pharmaceuticals with nitrogen active sites. Most of the studies that focus on the nitrification/denitrification process for endocrine disruptors removal, such as estrone (E1), estriol, 17β-estradiol or 17α-ethinylestradiol, showed that it was possible to eliminate up to 90% of natural and synthetic hormones within a few hours [72,73,74]. It is possible to extend the use of nitrogen removal processes to a wide range of pharmaceuticals with variable results regarding their high biodegradation potential [32,75,76].

**Figure 5:**
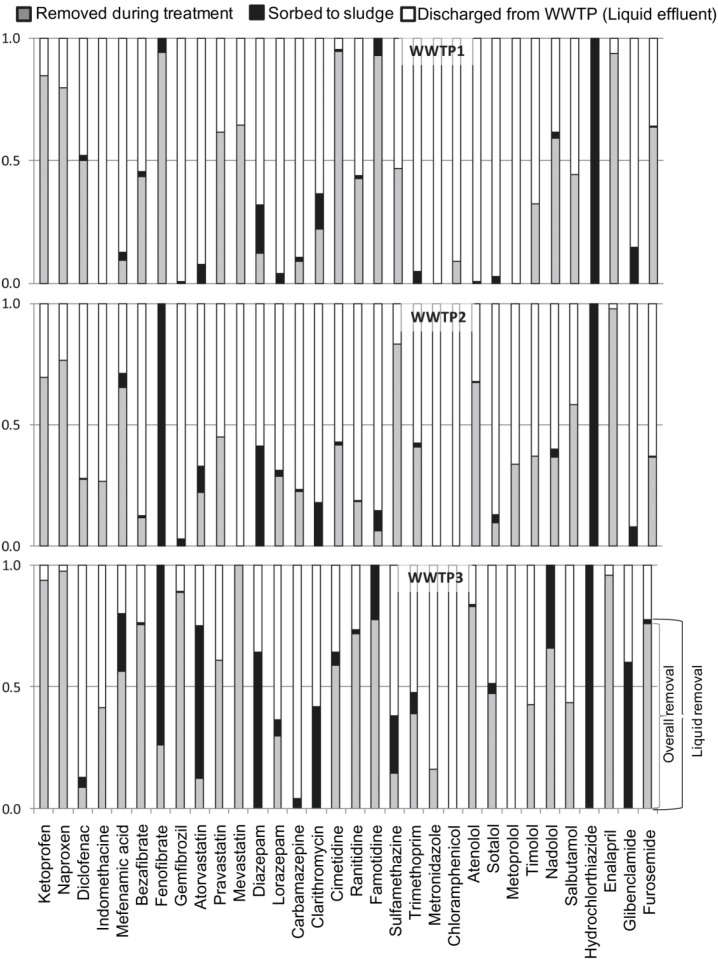
Quantity of pharmaceuticals degraded, adsorbed on sludge and discharged in the effluent. From Jelic *et al*. 2011 [77], with permission from Elsevier.

The mechanism of biodegradation of micropollutants depends on the compounds and on the bacteria species. However, it has been demonstrated that large amounts of pharmaceuticals are adsorbed on sludge, whereas some of them will be more or less degraded by the bacteria [77]. Some molecules will be adsorbed more easily (Figure 5), thus a lot of care has to be taken when sludge is recycled, for example, for agricultural purposes.

However, in the study reported by Gao *et al*. [78], it is reported that the depletion of pharmaceutical compounds (acetaminophen, caffeine, carbamazepine, chlortetracycline, demeclocycline, doxycycline, erythromycin, lincomycin, meclocycline, oxytetracycline, sulfadiazine, sulfamerazine, sulfamethazine, sulfamethoxazole, tetracycline and tylosin) was mainly caused by biological transformation (from 22% to 99%), while the sorption into sludge contribution was relatively irrelevant (7%).

Many other studies have reported the incidence and removal efficiencies of a broad spectrum of PPCPs in wastewater treatment plants (WWTPs) of different countries [26,43,58,78,79]. All of the studies concluded that the effectiveness of the conventional treatment for the PPCPs’ depletion was dependent on the chemical structures and physiochemical properties of the contaminants, as well as on the specific treatment processes of every WWTP.

Meteorological conditions, the presence of inhibitors and process conception (effluent, retention time, other treatments) can lead to changing degradation rates [80]. For aerobic treatment, increasing temperature is not beneficial, because it has a negative impact on oxygen dissolution in water. Water pH modifications can also inhibit the action of biomass. Actually, the major parameter that can affect the biodegradation performance is the retention time. Even if Carballa *et al*. [70] reported that temperature and a long retention time do not have a huge impact on the degradation yield in anoxic conditions, activated sludge systems need high retention times compared to other processes, like ozone or advanced oxidation processes [81,82,83,84].

Indeed, biological treatments rarely allow the total depletion of all wastewater pollutants, and even though the effluent quality may match the regulation, some recalcitrant compounds can be still present in treated wastewaters at very low concentrations and then be still dangerous for human health. For example, a recent work reports that the occurrence of very low concentrations of antibiotics (*i.e.*, sub-inhibitory concentrations) in wastewaters may favor the apparition and dissemination of bacterial resistance [85]. Consequently, it is needed to add a complementary treatment to improve the water quality before disposal.

## 3. Membrane Bioreactors (MBR)

**Figure 6:**
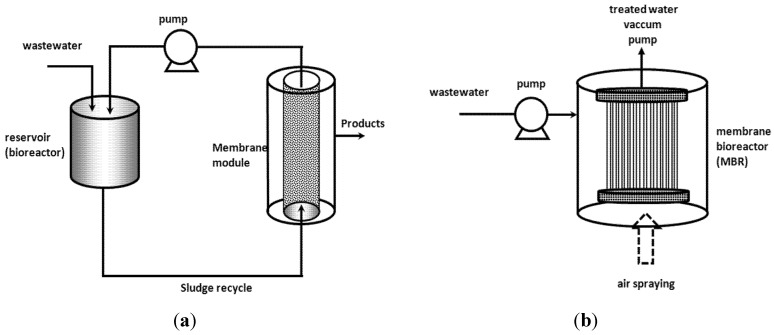
Two possible configurations of a membrane bioreactor (MBR). (**a**) Separated bioreactor and membrane unit; (**b**) bundle of hollow-fibers or assembly of flat membranes submerged into the bioreactor.

Membrane bioreactors (MBRs) combine biodegradation with a separation step to retain sludge (suspended solid) in the system for higher pharmaceuticals removal. These reactors can be composed of two units: a bioreactor tank and a membrane module, but generally, these two units are combined in only one, where the membrane bundle of hollow fibers or an assembly of flat membranes are submerged inside the bioreactor (Figure 6).

The possibility of uncoupling the hydraulic and sludge retention time (HRT and SRT) in tangential filtration is a clear advantage with respect to traditional gravity settling [86], as it allows MBRs to achieve a high sludge retention time (SRT) within compact reactor volumes, which is a great improvement in comparison to conventional AS systems. Indeed, they can achieve better degradation yields than classical AS processes. However, a continuous aeration in the lower part of the membrane bundle is generally necessary in order to ensure the saturation in oxygen, while creating enough turbulence to decrease as much as possible the membranes’ fouling and clogging, which are the major drawbacks of such processes. A detailed study case of the operating advantages and drawbacks of an MBR has been recently reported by Kaya *et al*., 2013 [87], who reported the depletion of etodolac, a nonsteroidal anti-inflammatory drug. They demonstrated that the etodolac depletion diminished from 80% to 27% with the decrease of the SRT from 30 to 15 days. At the same time, they observed a dramatic decrease of the permeate flux from the initial to steady state flux (approximately five- to 10-times) during the process for both SRT.

**Figure 7:**
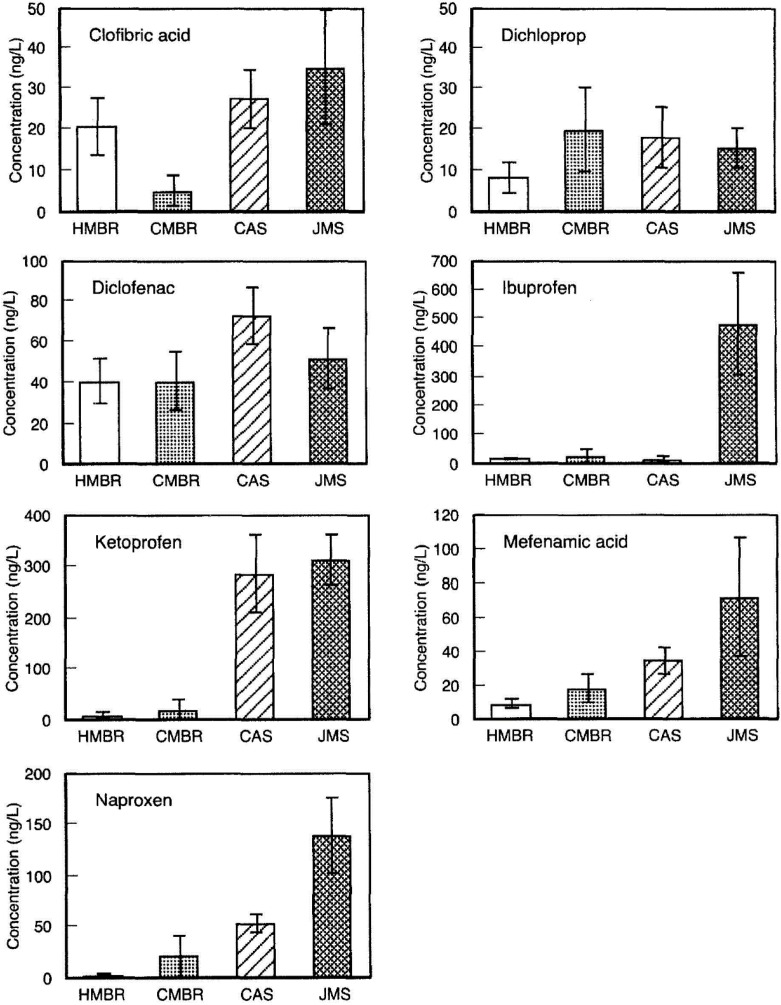
Comparison of removal efficiency of MBR and activated sludge (AS). HMBR, hybrid membrane bioreactor; CMBR, conventional membrane bioreactor; CAS, conventional activated sludge; JMS, jet mixed separator (coagulation/sedimentation). From Kimura *et al*., 2005 [90], with permission from Elsevier.

Clara *et al*., 2005 [88], analyzed the degradation of different pollutants, including pharmaceuticals, fragrances and EDCs, in several WWTP. For this purpose, they operated a pilot scale MBR at diverse SRT, and the results were compared with those of the AS systems. They concluded that some compounds, like carbamazepine, are really refractory to decomposition in the MBR, even at high SRT, whereas others, like ibuprofen, were removed by more than 90%. MBR and AS processes have been applied for the removal of some poorly persistent polar contaminants, like diclofenac, mecoprop and sulfophenylcarboxylate, by Bernhard *et al.* [89]. They determined that the MBR allows obtaining significantly better depletion. Other interesting works have been performed to compare both biological treatments [90,91,92,93]. These authors reported a better removal of some certain pharmaceuticals and similar degradation rates for the others in the case of MBR (Figure 7). However, like in the work of Clara *et al*., 2005, they observed that carbamazepine, which is a hardly-degradable micropollutant, is not depleted at all.

Radjenovic *et al*. [94,95] have reported the degradation of different pharmaceutically active compounds using two pilot-scale MBRs. The first MBR was equipped with a bundle of hollow-fiber ultra-filtration membranes, whereas the other MBR was operated with a micro-filtration flat-sheet membrane module. In most of the cases, they concluded that MBR is more efficient than activated sludge for the removal of antibiotics and EDCs, because the membrane can filter suspended solids with adsorbed pollutants [95]. However, membrane fouling can occur and needs to be regulated by mixing or air injection. In addition, pH has to be controlled to optimize the biodegradation. As is the case in AS, adsorption occurs also in MBR, and sometimes, the depletion observed is a mixed contribution of both processes: adsorption and degradation. The relative contribution of both processes has been recently studied by Fan *et al*., 2014 [96]. For this purpose, they conducted experiments with a sterilized sludge and with an AS to study the relative contributions of biodegradation and sludge adsorption for the depletion of acetaminophen, 17β-estradiol, naproxen, diclofenac and carbamazepine in a submerged MBR. They concluded that diclofenac removal mainly resulted from adsorption, whereas for the other pharmaceuticals tested, the biodegradation coupled with adsorption were responsible of the depletion observed (up to 98% for 17β-estradiol).

**Figure 8:**
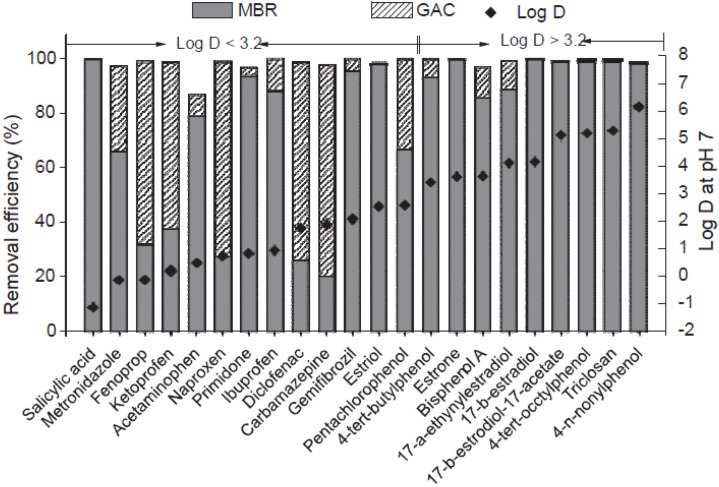
Complementary treatment of MBR with granular activated carbon (GAC). From Nguyen *et al*., 2012 [97], with permission from Elsevier.

Hence, MBRs can be coupled to other complementary treatments with a positive impact on traces of compounds not fully removed by biodegradation [97,98,99]. Figure 8 shows that complementary treatment with granular activated carbon (GAC) may be a promising solution for high overall removal of reluctant pharmaceuticals.

Other authors have imagined original configurations of the MBR in order to enhance the degradation of refractory micropollutants. Chen *et al*., 2014 [100], reported the application of a novel MBR with a multi-sparger multi-stage airlift loop for the depletion of 7-aminocephalosporanic acid in wastewaters. They reported that their system presents some advantages, like a better volumetric mass transfer coefficient and higher gas holdup, while having less mixing time than a single-stage loop reactor. More recently, Dutta *et al*., 2014 [101], reported the application of a two-stage anaerobic fluidized membrane bioreactor with two MBRs for the treatment of municipal wastewater. Granular activated carbon was used as the support medium for the microorganisms, but also as the adsorbent for the pharmaceuticals. They reported that a relatively good efficiency (90%) for the depletion of some pharmaceuticals, like sulfadiazine, ciprofloxacin and naproxen, and even the very recalcitrant carbamazepine, can be reached simultaneously with the removal of 95% of the chemical oxygen demand (COD). However, this result cannot be only attributed to the biotransformation, but also to the adsorption onto activated carbon and biofilms. As is usual in anaerobic processes, biogas was also produced.

The efficiency of biological treatments, as well as MBRs to deplete some recalcitrant pharmaceuticals is related to the ability of some microorganisms or consortia to metabolize or to become resistant to these molecules. Three major mechanisms are displayed by microorganisms to develop active drug resistance: efflux of the drug from the cell, the modification of the compound target or by the synthesis of enzymes able to selectively target and destroy the action of such compounds; some of these mechanisms are relatively well known in the particular case of antibiotics [102]. Indeed, bacteria are able to develop resistance to antibiotics; it is particularly the case of microbial consortia, which become resistant to erythromycin [103]. Erythromycin-resistant bacteria, in fact, have developed mutations that allow the starting of the synthesis of erythromycin esterase, an enzyme able to degrade this antibiotic. Actually, this property is used for the bio-production and isolation of this enzyme [104].

Moreover, the bioprocesses described above can be not well adapted for the treatment of some industrial effluents, which usually contain a relatively high concentration of pharmaceuticals, because some of these active molecules can obviously inhibit some metabolic ways of microorganisms, be toxic or even completely destroy the bacteria flora.

The drawbacks of the biological treatment of effluents described above have encouraged some research groups to work on the direct biodegradation of pollutants, but not with whole-cells, but with enzymes, which are one of the biochemical ways used by microorganisms for degradation. In the next section, the treatment of wastewaters by an enzymatic treatment, including the enzymatic membrane reactors, will be described.

## 4. Enzymatic Treatments

### 4.1. Biocatalysts

For the last two decades, the application of enzymes for environmental remediation has been widely studied [105,106,107,108,109]. Indeed, the use of enzymes instead of microorganisms for pollutant depletion from waters has several advantages. In fact, these biocatalysts, which are not a biological living system, but a biochemical one, are able to reach very high reaction kinetics for the degradation within mild conditions (temperature, pH) without being affected by the biological activity of the targeted compounds.

According to Demarche *et al*. [106], oxidoreductases EC1 (*i.e.*, peroxidases, polyphenol oxidases (PPO)), hydrolases EC3 (*i.e.*, proteases, esterases, lipases and cellulases) and lyases EC4 are suitable for wastewater treatment applications. Hydrolases can treat biological wastes, while oxidoreductases are good candidates for the detoxification of textile effluents or wastewaters containing phenols, aromatic compounds or hormones [110]. The use of a laccase, a lipase or a cellulase or an association of these enzymes can permit the inactivation of antibiotics in wastewater effluents, thus preventing the pollution of the environment [111].

Enzymes, like oxidoreductases (*i.e.*, peroxidases and laccases), are able to react with a large spectrum of substrates. These extracellular enzymes, generally extracted from ligninolytic fungi, are very efficient in PPCP removal. [105,112].

Peroxidases, which degrade some substrates in the presence of H_2_O_2_, are able, in proper conditions (optimal pH, temperature, H_2_O_2_ concentration, enzyme/substrate ratio, *etc*.), to achieve 80% or higher removal of natural and synthetic hormones from synthetic waters within 1 h of treatment [113,114,115]. However, lower degradation rates (about 30%) were obtained in actual wastewater samples, because of the negative impact of other organic compounds inhibiting the enzyme activity or competing for H_2_O_2_ or horseradish peroxidase (HRP) oxidation sites [116]. Peroxidases also proved to be active in removing PPCPs, such as triclosan [117], diclofenac [118] and tetracyclines [119]. Between 70% and 99% of tetracycline antibiotics can be eliminated during a 4-h treatment [120,121]. Nevertheless, the degradation was very limited for other reluctant substrates, like carbamazepine [122]. In addition, as explained above, peroxidases need the presence of H_2_O_2_ as a co-substrate to initiate the degradation reactions, unlike laccases, which catalyze the oxidation of some aromatic compounds and, more specifically, phenols, simply using the dissolved O_2_ as the electron acceptor [105,123,124].

The potential of laccase-catalyzed reactions was investigated and successfully used in research and industry for the synthesis or removal of persistent pollutants [121,125,126,127,128]. As ligninolytic enzymes, laccases can be used for the detoxification of highly concentrated wastewater from the forest product industry [129]. However, laccases also proved to be very efficient for nearly complete depletion of estrogens (natural or synthetic) from water within 1 h of reaction [115,130,131]. These enzymes present also some activity towards antibiotics, like tetracycline, chlortetracycline, doxycycline and oxytetracycline, which have been degraded without adding any chemicals by 16%, 48%, 34% and 14% after 4 h of reaction, respectively [121]. Their efficiency towards reluctant anti-inflammatory drugs’ removal depends on the compounds and the biocatalyst origin. Margot *et al*. [132] showed that 25% of diclofenac and 95% of mefenamic acid could be depleted in 20 h with laccase from *Trametes versicolor*, whereas Lloret *et al*. [131] reported that laccase from *Myceliophthora thermophila* could degrade up to 65% of the diclofenac, but was ineffective towards naproxen.

To sum up, peroxidases and laccases can successfully remove hormones and phenolic pollutants with equivalent results [114,115], even though laccases seem more interesting, because they do not need the addition of H_2_O_2_ and are less affected by other organic pollutants in wastewater [133]. Nevertheless, the activity of laccases towards non-phenolic pharmaceutical compounds is not very important, but can be significantly enhanced by adding a redox mediator in the reaction medium [121,124,134,135,136,137,138].

**Figure 9:**
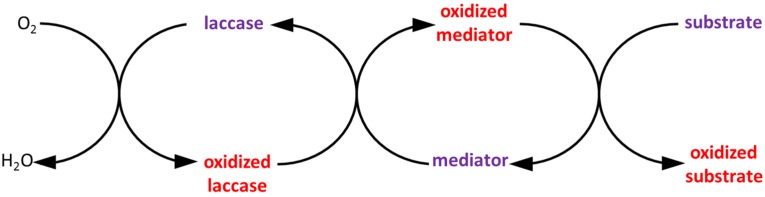
Catalytic cycle of a laccase with a mediator of oxidation. Adapted from Banci *et al*., 1999 [139].

Indeed, redox mediators can react with the enzyme to create very reactive intermediates, which will degrade the targeted substrate and then be regenerated for a new cycle (Figure 9).

Many natural and synthetic mediators, such as 2,2'-azino-bis(3-ethylbenzothiazoline-6-sulphonic acid) (ABTS), acetosyringone, syringaldehyde, vanillin or violuric acid, allow increasing the laccase or peroxidase degradation potential [117,119,120,131,134,140]. Their use allowed the depletion of antibiotics, such as tetracyclines [119,120,121] or sulfonamides [134], by increasing the reaction rate up to 80-times with respect to the baseline degradation rate and degrading them almost completely in one hour. Similar results were obtained for the treatment of estrogen hormones [114,131], triclosan [117,141] and other pharmaceuticals, such as oxybenzone [140] or even the refractory carbamazepine [122]. Moreover, the addition of a mediator allows reducing the amount of enzymes needed for the successful removal of estrogens [114,130]. Nevertheless, the addition of redox mediators could not be an economically viable option for continuous wastewater treatments; it seriously increases the cost and may lead to increased toxicity, due to the difficulty of removing them from the effluent.

### 4.2. Enzymatic Reactors

Even if enzymatic catalysis has shown at the industrial scale its capacity to achieve, under mild conditions, complex reactions with high selectivity and conversions, the choice of this technology for environmental remediation needs consequent efforts in research and development, because an important gap still exits to ensure the economic and technical viability of this process at the industrial scale. Batch reactors with free enzymes may not be an economically viable solution for wastewater treatment; the volumes to be treated are enormous, as well as are the enzyme quantities to be used for this purpose. In addition, at the end of the process, enzymes need to be removed from the effluent and be treated like waste. As far as enzymes are relatively expensive, the global economic viability of the process should be demonstrated. Indeed, for industrial-scale requests, using immobilized enzymes appears to be essential in view of the reuse of the biocatalyst, then decreasing the cost, while allowing a continuous process. In addition, enzyme immobilization generally results in an enhancement of the biocatalyst stability regarding the temperature, pH, organic solvents or storage [142,143], even if, sometimes, a loss of activity has been observed. Immobilization of enzymes could also allow increasing the surface of contact between enzymes and substrates, avoiding too much shear stress due to mixing, which could inactivate enzymes, and maintaining a good catalytic efficiency over many reaction cycles [124,144,145].

Processes with enzymes grafted on a support represent an interesting option to degrade the reluctant pollutants, which are not completely eliminated during classical wastewater treatment, while decreasing the processing costs by reusing the biocatalyst [145,146,147]. Enzymes are generally immobilized on particulate solids via different technics, such as adsorption, entrapment, encapsulation or covalent bonding [145,147,148,149,150,151,152,153]. Among the immobilization methods available, covalent bonding formation on carriers seems very promising for industrial applications. This enables avoiding the leaching of enzymes with a strong link between the enzyme and the support, making it possible to degrade a large range of pollutants, especially with laccases [154,155,156]. Besides conventional immobilization on solid supports, cross-linking enzyme aggregates (CLEAs) also allow one to enhance enzyme stability [152]. Recently, laccase-grafted particles, as well as laccase CLEAs have been efficaciously used for the depletion of EDCs in fixed-bed reactors [157,158] or in fluidized-bed reactors [142,143,159,160]. However, those kinds of reactors fall outside of the scope of this review, which aims to focus on membrane reactors, as previously asserted in the Introduction.

## 5. Enzymatic Membrane Reactors (EMRs)

**Figure 10:**
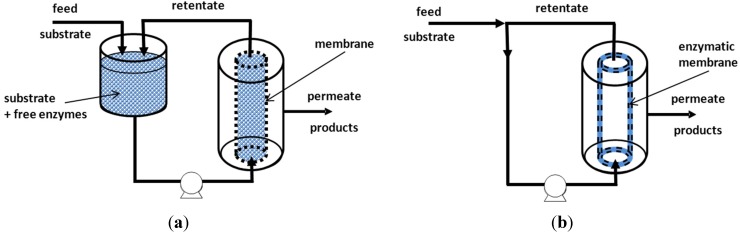
Enzymatic membrane reactors (EMRs). (**a**) Enzymatic reactor coupled with filtration unit: the membrane is only used as a selective barrier; (**b**) enzymatic membrane reactor (the membrane acts as a selective barrier and biocatalyst support).

According to the role played by the membrane, Sanchez and Tsotsis [161] and Jochems *et al*. distinguished two types of enzymatic membrane reactors (EMRs) (see Figure 10) [148]. In the first case (Figure 10a), the enzymatic reactor is associated with a filtration unit, and the membrane acts as a barrier; it retains the biocatalysts inside the reactor throughout the process, while reaction products are transferred through the membrane. Actually, only the second case (Figure 10b) corresponds to a genuine enzymatic membrane reactor. In such a reactor, the membrane acts as a selective barrier, and at the same time, it is the support of immobilized enzymes. The reaction takes place where the biocatalyst is immobilized: at the external or internal surface of the membrane or inside the porosity and during the transfer through the membrane. This configuration has many advantages, as it provides enzyme stability by immobilization and reduces the external or internal diffusion phenomena present on a classical porous support. Another advantage of EMRs is the fact that the substrates are forced to approach the biocatalytic sites during filtration process; this concept, called “flow through membrane reactor”, is being considered as the main benefit of this process intensification [161]. Moreover, both of the configurations presented in Figure 10 have been explored for the depletion of recalcitrant pollutants from wastewater.

### 5.1. Enzymatic Reactor Coupled to a Membrane Unit

In such EMRs called stirred-tank membrane reactors (STMRs), the substrate is continuously fed into the reactor, where enzymes have been previously added to the reaction medium. The reaction mixture is flowed in the tangential direction along the membrane and then is recycled into the reactor. In this configuration, separated reaction and separation devices are placed in series and, then, can be controlled independently: the variation of the reactor volume and membrane surface, as well as the operating conditions, like temperature, pH, substrate, biocatalyst concentration, fluxes, HRT and pressure, allow optimizing the whole process straightforwardly. According to Nguyen *et al*. [162], the removal of diclofenac after a contact of 8 h in a batch reactor was only 30%, while a removal of 60% was achieved during continuous operation of the EMR at an HRT of 8 h. One drawback of this configuration concerns the value of tangential flow required to avoid as much as possible the polarization of concentration and to ensure a feasible filtration rate; if the tangential flow is too high, it can result in a high shear stress and then inactivates the enzymes.

In this configuration, the choice of the membrane is crucial in terms of cut-off. The right membrane has to be selected in order to guarantee the retention of enzymes, as well as the substrate; but the membrane pores must be large enough to allow the product to pass to the permeate. Historically, stirred-tank membrane reactors have been investigated for hydrolysis reactions, which involving large amounts of substrate and leading to small products [163,164,165,166], but such reactors were also investigated for environmental applications (see Table 2). Recent studies have focused on the application of an enzyme STMR for the removal of polyphenols or dyes [167,168] and estrogen or EDCs [169,170]. For the depletion of bisphenol A (BPA), diclofenac, carbamazepine, sulfamethoxazole and atrazine, Nguyen *et al*. [162,171] proposed submerging a hollow fiber module into the bioreactor. In that case, as the biocatalyst is not recirculated, denaturation due to the shear stresses is avoided, and the catalytic potential of enzymes is thus preserved.

**Table 2:** Free enzyme membrane reactors for waste water treatment.

Enzymes	Membrane Type	Reactor Type	Applications	Ref.
Laccase from *C. bulleri*	PAN UF membrane 20 kDa	CSTR	Degradation of triarylmethane dyes	[168]
Tyrosinase	PES UF membrane 30 kDa	CSTR	Degradation of polyphenols	[167]
Laccase from *T. versicolor*	PS UF Membrane 10 kDa	CSTR	Degradation of dyes	[172]
laccase from *M. thermophila*	PES UF membrane 10 kDa	CSTR	Degradation of estrogen	[157]
Laccase and HRP	Flat sheet polymeric NF membranes	CSTR	Degradation of BPA	[170]
Laccase from *A. oryzae*	6 kDa polyacrylonitrile hollow fiber membrane	Membrane submerged in the reactor	Degradation of BPA and diclofenac	[171]
		Membrane submerged in the reactor; GAC was added	Degradation of carbamazepine, diclofenac, sulfamethoxazole and atrazine	[162]

Notes: PAN, polyacrylonitrile; UF, ultrafiltration membrane; PES, polyethersulfone; PS, polysulfone; BPA, bisphenol A; GAC, granular activated carbon; CSTR, continuous stirred tank reactor.

All of these studies have concluded the feasibility of the continuous degradation of reluctant pollutants. However, even if the authors have chosen membranes with an adequate cut-off in order to avoid the loss of enzymes in the permeate, a gradual decrease of the enzymatic activity has been generally reported, indicating that enzyme denaturation occurs during this continuous operation. This decrease of the enzymatic activity can be caused by a normal activity decay of free enzymes or by denaturation due to shear stresses, as explained above. The decay of the enzymatic activity in such EMRs has been sidestepped by Nguyen *et al*. [162,171] by adding periodically a dose of the commercial laccase solution during several days in order to maintain an interesting degradation rate of carbamazepine and other pharmaceutical products. Nevertheless, this continuous feed of enzymes could be discarded from an economical point of view. Gasser *et al*. [173] report a more interesting solution: the use of laccases immobilized onto silica nanoparticles instead of free enzymes. They observed an efficient removal of BPA (75%), all along a period of 45 days.

Furthermore, as has been described earlier for classical enzymatic reactors, the addition of redox mediators, like ABTS, enhances the removal of pollutants, like dyes [168]. In the work of Nguyen *et al*. [162] described above, they studied also the removal of pharmaceutics, like carbamazepine, diclofenac, sulfamethoxazole and even atrazine, using the syringaldehyde as the mediator. However, as far as the molecular weight and size of these mediators are relatively low, they were not retained by the membrane used in this work. Indeed, it is difficult to imagine an industrial process with mediator addition, even though some authors have reported their recycling. Chhabra *et al*. [168] suggested that ABTS can be recovered from permeate by precipitation with a solution of ammonium sulfate. Nguyen *et al*. [162] used another strategy: they added the syringaldehyde together with granular activated carbon (3 g·L^−1^) to a solution of pharmaceuticals. The concomitant addition of both products resulted in an enhancement of 14%–25% for the removal of the pharmaceuticals named above. They confirmed that the improvement observed was not due solely to adsorption, but also due to enhanced biodegradation, thanks to a mass balance analysis.

As has been stated above in the first part of this section, the use of membrane reactors offers some drawbacks: besides the problem of enzyme deactivation by shear stress, membrane fouling has been generally reported. Actually, in order to retain the biocatalyst, most of the studies have been carried out with ultrafiltration membranes (UF) made of polyethersulfone (PES) or polyacrylonitrile (PAN) with cut offs between 6 and 30 kg·mol^−1^. Other authors have reported the use of a nanofiltration (NF) unit [170] or more exceptionally submerged 0.2-µm membranes [162,171,173]. If UF membranes are able to retain the enzymes, they also retain polymeric products, resulting in the oxidative coupling reactions of some phenolic substrates. By the action of the pressure gradient, these polymeric products can be accumulated on the membrane surface, with the subsequent reduction of the permeate flow rate. A similar effect can be produced by the enzymes themselves, as they are proteins that can form a dynamic gel layer on the membrane surface, resulting in a flux decrease or a transmembrane pressure enhancement, which finally reduces the cost-efficiency of the process. The use of EMRs in Configuration B shown in Figure 10, where enzymes are immobilized at the membrane surface or within the membrane porosity, could be a solution for reactions resulting in polymeric products. Indeed, in such a case, the oxidation occurs while the reactants are forced-flowed through the membrane, and then no-polymerization occurs on the retentate side, thus limiting the membrane fouling. This type of EMR is presented below.

### 5.2. The Genuine Enzymatic Membrane Reactor

**Figure 11:**
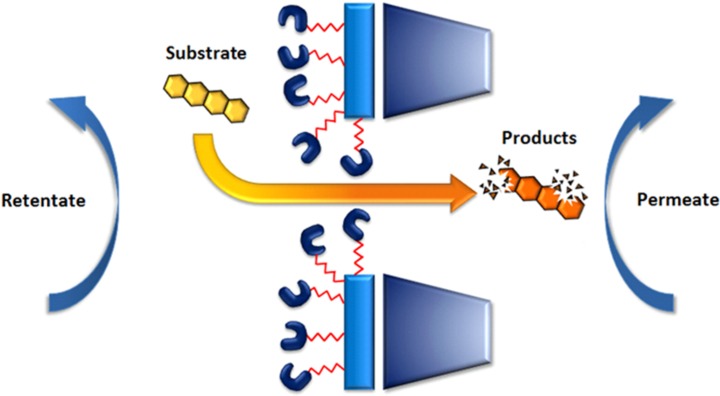
Enzymatic degradation within the pores of a membrane.

In genuine EMRs, the biocatalyst is retained within the membrane itself, and the contact between the substrate and the enzyme occurs during the mass transfer process result of the transmembrane pressure. Indeed, the reaction takes place simultaneously with the mass transfer process through the membrane, and products are collected in the permeate (see Figure 11).

This special configuration results in better control of the reaction through the “micro-reactor concept”, where the distance between the catalyst and the substrate is reduced considerably, then increasing the probability of reaction. Indeed, the membrane can be considered as a specific macro-system resulting from the assembly of pores, micro-systems or micro-reactors. In such a system, the contact between the molecules of the substrate and biocatalysts is improved as the mass transfer path is reduced, while the contact time can be controlled through the mass transfer rate. Theoretically, with this special configuration, a good choice of the membrane and process parameters allows one to expect process optimization, with good control of the reaction kinetics, contact time and reduced losses of substrate and catalyst, resulting in higher yields and cleaner products [174]. Moreover, the energy costs of such EMRs should be reduced in comparison with a classical packed bed reactor, because the membrane thickness and pressure drop are much smaller. Another advantage of EMRs is that these processes are modular, and their scaling up is very simple. Moreover, in cases where the conversion in one step is not very high, recycling or a configuration with various EMRs in series can be easily implemented. Nevertheless, the process optimization needs to find a balance between mass transfer through the membrane and enzyme kinetics [148].

**Figure 12:**
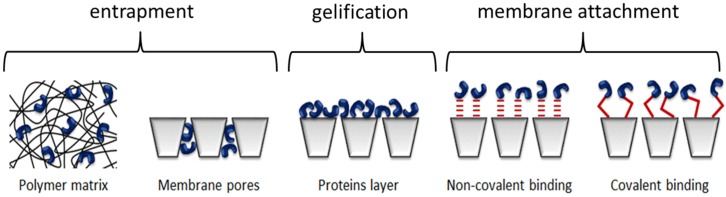
Diverse types of enzymatic membrane preparation adapted from [175].

The choice of enzyme immobilization method depends on the characteristics of the membrane (material, properties, *etc*.), the chosen enzyme properties (activity, stability, working conditions resistance, temperature, pH, solvent), the advantages of the method (a process easy to be carried out, strong bonding, *etc*.) and also the cost (enzyme, products for immobilization process, membrane regeneration, final product value) [148]. Whatever the working conditions are, one of the most important points is the appropriate integration of the biocatalyst on or within the membrane. As seen in Figure 12, there are three main methods for active membranes preparation: entrapment within the membrane porosity, deposition of a gel layer of enzymes on the membrane surface and attachment through covalent or non-covalent bonds on the membrane [148].

The entrapment of enzymes within a membrane structure can be accomplished either during membrane elaboration or by filtering an enzymatic solution. In the first case, the enzymes are mixed with a polymeric solution before membrane conditioning. The biocatalyst can be simply physically entrapped or it can be covalently linked to the polymer matrix to avoid enzyme leakage [176]. In order to reduce enzymes loss during the reaction, it is possible to add a bifunctional agent, like glutaraldehyde, in the mix to create a covalent bonding between enzymes and the polymer and then create aggregates [177,178]. Enzymes can also be attached on a solid support before mixing them with the polymeric solution [179]. Whatever the chosen solution is, these methods of active membrane preparation are not the most interesting, because the membrane cannot be regenerated when the enzymes lose their activity. This drawback has been bypassed by other researchers who have developed another method that is simpler and more widespread [180,181,182]. They filtered the enzyme solution through the membrane in order to retain them within the porosity of the membrane support. This method increases the quantity of immobilized enzymes, but also the risk of enzymes leaching. However, it is possible to prevent loss by forming enzymes clusters inside the membrane pores.

Enzymatic membranes can also be prepared by creating a gel of enzymes on the membrane surface. This method is based on the well-known protein ability to form a gel on membrane surface during filtration. For this purpose, an enzymatic solution is filtered through a membrane (UF or MF), and the retained proteins create a continuous gel film on the filtration surface [183,184]. In this case, the biocatalyst film is not firmly attached to the membrane surface with covalent bonds, and then, the risk of enzyme loss is relatively important. However, this method is very interesting, because when the enzymes lose their activity, membranes can be easily regenerated and a new enzymatic layer formed again. The stability of the enzymatic layer can be improved by creating covalent bonding between enzyme molecules using a di-aldehyde, like glutaraldehyde [182]. This immobilization method is certainly easy to carry out and results generally in interesting immobilization ratios. However, as in the case of physical entrapment, it is possible that the totality of enzyme molecules are not available or even active because of diffusion limitations or steric hindrance. Enzymes can also be tied to the surface by non-covalent bonding with low energy interactions, such as Van der Waals interactions, hydrophobic interactions, hydrogen bonding, ion bonding, charge transfer or chemisorption [148]. The greatest advantage of this method is its simplicity. It is a cheap one-step process, because there is no need to add any activating agent. Although it has been reported that protein adsorption is enhanced on hydrophobic surfaces and then membranes [185], there is a high risk of enzymes desorption due to temperature or pH changes, because of the bonding weakness, which can reduce the process efficiency [186].

In order to limit enzyme leaching, which can sometimes be observed when the immobilization methods described above are applied, covalent bonding has been used to strengthen the enzyme-membrane links. The formation of covalent bonding between enzymes and the functional groups on the membrane surface can be carried out using diazonium salts, carbodiimides, cyanogen bromide, glutaraldehyde or epichlorohydrin [175]. The use of bifunctional agents, such as glutaraldehyde, has been broadly reported [128,187,188,189,190]. These agents can indeed stabilize the biocatalyst regarding environmental factors [144,191]. It is also possible to enhance the immobilization efficiency by functionalizing the membrane with TiO_2_ [192]. Laccase and catechol oxygenase covalent bonding with glutaraldehyde for immobilization on nylon and polyamide membranes has already been carried out for phenolic compounds degradation [190,193,194,195]. Covalent bonding immobilization enhances enzymes’ stability, while avoiding biocatalyst leaching, particularly in non-aqueous reaction media. Unfortunately, when the enzymes lose their activity, the irreversibility of this grafting method may become a drawback, because the carrier regeneration is more difficult than with a simple adsorption. Other disadvantages can be considered, as the immobilized enzymes by this method can present lower activity when compared with free enzymes if they are immobilized in an inactive conformation or if the active site is involved in the bonding reaction. This method is interesting for the cases where there is a small amount of denaturized enzymes or the support can be regenerated easily [175]. Moreover, the covalent bonding immobilization is expensive, due to the cost of chemicals for carrier activation. In order to avoid this issue, Belleville *et al*. [187] developed a simple method to functionalize ceramic membranes in order to immobilize enzymes. The porous support is first hydrated and then coated with a biopolymer layer by filtering a gelatin solution. In the next step, this layer is cross-linked and activated with a glutaraldehyde solution. Bonds are created between gelatin free -NH_2_ groups and glutaraldehyde = CO groups. Finally free = CO groups of glutaraldehyde react with enzyme free -NH_2_ groups in the last step of the process. This method was used to successfully immobilize proteases [196], lipases [189] and laccases [128]. The advantage of this method is the possibility of cleaning the membrane support when the enzymatic activity decreases, a fact that allows the preparation “*in situ*” of a new enzymatic membrane on the same support.

Most of the enzymatic membranes presented in the literature for environmental applications concerns active membranes prepared with lipases, laccase or, more generally, oxidoreductases. However, these enzymatic membrane reactors have been studied generally for the depletion of some pollutants, like phenols or related compounds; only one very recent example has been reported in the literature for the degradation of pharmaceuticals and, more particularly, of tetracycline [197] (see Table 3).

**Table 3:** Oxido-reductase grafted membranes and their applications in wastewater treatment.

Type of immobilization	Enzymes	Membrane Types	Immobilization Types	Applications (removal of)	Ref.
**Entrapment**	Crude enzyme extract of *Pseudomonas sp.*	Flat polyacrylonitrile (PAN) UF membrane	Entrapment within membrane by mixing the enzymes with casting solution	phenols	[198]
	Laccase from *P. oryzae*	Spira-cel spiral wound module with a polyethersulfone membrane	Entrapment within membrane by filtration	phenols	[199]
	Laccase and horseradish peroxidase	Polypropylene hollow fiber membrane (0.2 µm)	Entrapment within membrane by filtration	hydroxylated aromatic compounds	[200]
	Laccase from *Trametes versicolor*	TiO_2_ blended polyethersulfone (PES) membranes and TiO_2_ sol-gel coated PVDF membranes (0.1 and 0.45 µm)	Adsorption or covalent bonding on TiO_2_ nanoparticle	Bisphenol A (BPA)	[192,201]
**Membrane attachment**	Polyphenol oxidase	0.45 µm flat nylon membrane and polysulfone capillary membrane	Adsorption with glutaraldehyde cross-linking	phenols	[202]
		Polyethersulfone and polysulfone capillary membranes	Adsorption	phenols	[203,204]
		Polyethersulfone capillary membranes and hydrophilic nylon flat-sheet membranes	Adsorption or adsorption with glutaraldehyde cross-linking	p-cresol	[205]
	Crude enzyme extract of *Pseudomonas syringae*	Flat polyamide membrane (0.2 µm)	Covalent bonding	phenol and catechol	[193]
	Horseradish peroxidase	Flat polyacrylonitrile (PAN) UF membrane	Adsorption and covalent bonding	phenol	[206]
	Laccase from *Trametes versicolor*	Flat modified PVDF microfiltration membrane	Covalent bonding	phenols	[207]
		Chitosan/poly(vinyl alcohol) composite nanofibrous membranes	Covalent bonding	2,4-dichlorophenol	[156]
		α-alumina membrane (0.2 and 1.4 µm)	Covalent bonding	phenols	[128]
				tetracycline	[197]

As shown in Table 3, these active membranes can be obtained either by enzyme entrapment or by covalent or non-covalent attachment on the membrane surface. In most cases, polymeric membranes were used, but inorganic membranes can also be employed [128,197]. Polymeric membranes enable direct covalent bonding of enzymes without using any activating chemicals, due to the fact that some polymers can present functional groups or be easily functionalized. However, as the mechanical, thermal and chemical resistances of polymeric membranes are quite low compared to those of inorganic ones, they cannot be easily regenerated when the biocatalyst becomes inactive. Similar problems have arisen when the biocatalyst is entrapped in a porous support. Moreover, as explained above, in spite of the obvious interest in the enzymatic treatment for the removal PPCPs from wastewater, most of the research has been focused on the use of a laccase-grafted membrane for the depletion of phenolic compounds present in some wastewaters. In such reactors, a high level of removal can be achieved, but the performances can be affected by membrane fouling [128].

There is only a recent work describing the potential of covalently grafted laccase on ceramic membranes for tetracycline antibiotic degradation in an EMR [197]. Grafted enzymes prove to be more efficient than free biocatalyst during batch tests for equivalent amounts of enzymes. It was demonstrated that the enzymatic reaction could be carried out in the reactor (119 mg of tetracycline degraded per hour and per m^2^) for more than 200 h without losing catalytic activity.

Each type of reactor has its own advantages and drawbacks (Table 4). For example, membrane reactors with free enzymes represent a viable solution when it is possible to carry out reaction and separation simultaneously. Enzymatic reactors with immobilized enzymes on beads or membranes seem to provide one of the most interesting configurations for enzymatic degradation of pharmaceuticals, because these allow the reuse of enzymes in a continuous process. They have been recently pointed as a very promising alternative for the depletion of such pollutants from wastewaters or even for groundwater treatment. However, the possibility of grafting on membranes is restricted to the limited surface or volume (porosity) of membranes, and then, the amount of grafted enzymes is relatively low. This limitation can be shortened by a very fast kinetics and/or employing several EMRs in series.

**Table 4:** Comparison of the advantages and drawbacks of continuous enzymatic processes.

Biological Treatment	Advantages	Drawbacks
Membrane reactor with free enzymes	Homogeneous mixing In some cases higher enzymatic activity than grafted enzymes	Less stability than with grafted enzymes
Packed-bed reactor with grafted enzymes	Biocatalyst recycling	Pressure drop Preferential pathways In some cases lower enzymatic activity than free enzymes Possibly diffusion limitations (internal and external)
Fluidized-bed reactor with grafted enzymes	Biocatalyst recycling Pressure drops reduced Better homogeneity	Additional energy cost (gas) In some cases lower enzymatic activity than free enzymes Possible diffusion limitations (internal)
Enzymatic membrane reactor	Biocatalyst recycling Decrease of diffusion limitations Separation and reaction take place simultaneously	Membrane clogging In some lower enzymatic activity than free enzymes In some cases lower enzymatic activity than free enzymes Limited loading of enzymes

Depending on the composition of wastewaters, preliminary treatments with activated sludge systems or classic membrane bioreactors may be needed. These previous treatments would eliminate the biodegradable part of the pollution, while refractory pollutants, like PPCPs and EDCs, would be treated in subsequent membrane or beads-based bioreactors. The enzymatic bioreactor process is an alternative solution to other types of refining treatments, like advanced oxidation processes or ozonation. Unlike them, enzymatic processes are generally environment friendly, as they do not rely on harsh chemicals and their energy consumption is relatively low.

Enzymatic membrane reactors would conveniently complement existing treatment facilities that are not equipped with advanced oxidation units. The envisioned enzymatic technology could lead to cheaper and greener processes, while allowing a full inactivation of the most worrying persistent and emerging pharmaceuticals in water effluents.

## 6. Conclusions

We reported in this review the most recent works related to the degradation of recalcitrant pharmaceuticals present in domestic or industrial wastewaters. Different types of bioprocesses and membrane reactors have been described: classical activated sludge (AS), MBR, enzymatic reactions and EMR. Under well-controlled operating conditions, the classical aerobic AS reactors can be relatively efficient for the removal of poorly biodegradable persistent pollutants: diclofenac, sulfophenylcarboxylates and antibiotics, like tetracycline or erythromycin, but rarely of hardly-degradable micropollutants, such as carbamazepine. However, MBR have been reported to be generally much more efficient than classical AS systems, because their performances can be optimized by varying separately the reactor volume or the membrane surface, as well as operating parameters, like temperature, pH, fluid velocity, pressure, enzyme and substrate concentration, as well as the separate control of different HRTs. It is important to notice that in both configurations (AS or MBR), the depletion rates observed are not only the result of the microbial metabolism, but also of the adsorption of the recalcitrant compounds in the sludge. Even if AS and MBR present a clear interest in the simultaneous depollution of organic matter, phosphates and other classical macro-pollutants together with recalcitrant micro-pollutants, they present some drawbacks. The first one is the final disposal of AS with adsorbed micro-pollutants. The second one is the adaptation of bacteria to some refractory pollutants, like antibiotics, which results in the development of the capacity to degrade the antibiotics and then to become antibiotic-resistant. MBRs can also be limited by clogging and fouling due to the accumulation and adsorption of polysaccharides, proteins or even biofilms on the membrane surface, but this limitation is partially solved at the industrial scale by using a frequent backwash.

The use of enzymes instead of microorganisms for pollutant depletion from waters has several advantages with respect to classical AS systems. In fact, enzymes are biocatalysts that are not affected by the biological activity of the targeted compounds, since they are not a biological system, but a biochemical one. For example, high concentrations of pollutants, which can be toxic to bacteria, can be treated. In addition, very high reaction kinetics for the degradation within mild conditions can be reached. Moreover, the enzymatic treatment of wastewaters present also some disadvantages; in fact, enzymes are very specific for some substrates and can be easily deactivated by concomitant pollutants in wastewaters. Nevertheless, both of these drawbacks can be partially solved if the enzymes are used for the treatment of some wastewaters from pharmaceutical industry or underground waters, which are relatively clean with respect to macropollutants. It is important to notice that enzymes are relatively costly, but this disadvantage can be circumvented by using immobilized enzymes on beads (stirred tank or fluidized bed reactors) or membranes (EMRs). These configurations are interesting for the enzymatic degradation of pharmaceuticals, because they allow the reuse of enzymes in a continuous process. Furthermore, EMRs present some other advantages, like the coupling of the filtration process with the biotransformation. Indeed, grafting enzymes inside membranes pores may be interesting, because the contact between the substrates and the biocatalyst is enhanced. However, EMRs present also some drawbacks. A minimal value of tangential flow is required to avoid as much as possible the polarization of the concentration and to ensure a feasible filtration rate, but if the tangential flow is too high, it can result in a high shear stress, which leads to enzymes inactivation. Moreover, the possibility of grafting on membranes is restricted to the limited surface or volume (porosity) of membranes, and therefore, the amount of grafted enzymes can be relatively low. This limitation can be shortened by a very fast kinetics and/or employing several EMRs in series. 

Only very few studies have been reported for the depletion of pharmaceuticals in EMR, even if these reactors would conveniently complement existing treatment facilities that are not equipped with advanced oxidation units. The envisioned enzymatic technology could lead to cheaper and greener processes, while allowing a full inactivation of the most worrying persistent and emerging pharmaceuticals in water effluents.

## References

[1] Patrick D.M., Marra F., Hutchinson J., Monnet D.L., Ng H., Bowie W.R. (2004). Per capita antibiotic consumption: How does a north american jurisdiction compare with Europe?. Clin. Infect. Dis..

[2] Al Aukidy M., Verlicchi P., Jelic A., Petrovic M., Barcelo D. (2012). Monitoring release of pharmaceutical compounds: Occurrence and environmental risk assessment of two wwtp effluents and their receiving bodies in the Po valley, Italy. Sci. Total Environ..

[3] Tambosi J.L., Yamanaka L.Y., Jose H.J., Moreira R.D.P.M., Schroder H.F. (2010). Recent research data on the removal of pharmaceuticals from sewage treatment plants (stp). Quim. Nova.

[4] Wirtz V.J., Dreser A., Gonzales R. (2010). Trends in antibiotic utilization in eight latin American countries, 1997–2007. Rev. Panam Salud Publ..

[5] Schwab B.W., Hayes E.P., Fiori J.M., Mastrocco F.J., Roden N.M., Cragin D., Meyerhoff R.D., D'Aco V.J., Anderson P.D. (2005). Human pharmaceuticals in us surface waters: A human health risk assessment. Regul. Toxicol. Pharm..

[6] Mompelat S., le Bot B., Thomas O. (2009). Occurrence and fate of pharmaceutical products and by-products, from resource to drinking water. Environ. Int..

[7] Kummerer K. (2009). Antibiotics in the aquatic environment—A review—Part I. Chemosphere.

[8] Li W.C. (2014). Occurrence, sources, and fate of pharmaceuticals in aquatic environment and soil. Environ. Pollut..

[9] Li D., Yang M., Hu J., Ren L., Zhang Y., Li K. (2008). Determination and fate of oxytetracycline and related compounds in oxytetracycline production wastewater and the receiving river. Environ. Toxicol. Chem..

[10] Larsson D.G.J., de Pedro C., Paxeus N. (2007). Effluent from drug manufactures contains extremely high levels of pharmaceuticals. J. Hazard. Mater..

[11] Chen F., Ying G.G., Kong L.X., Wang L., Zhao J.L., Zhou L.J., Zhang L.J. (2011). Distribution and accumulation of endocrine-disrupting chemicals and pharmaceuticals in wastewater irrigated soils in Hebei, China. Environ. Pollut..

[12] Siemens J., Huschek G., Siebe C., Kaupenjohann M. (2008). Concentrations and mobility of human pharmaceuticals in the world's largest wastewater irrigation system, Mexico city-mezquital valley. Water Res..

[13] Walters E., McClellan K., Halden R.U. (2010). Occurrence and loss over three years of 72 pharmaceuticals and personal care products from biosolids-soil mixtures in outdoor mesocosms. Water Res..

[14] Heberer T. (2002). Occurrence, fate, and removal of pharmaceutical residues in the aquatic environment: A review of recent research data. Toxicol. Lett..

[15] Deblonde T., Cossu-Leguille C., Hartemann P. (2011). Emerging pollutants in wastewater: A review of the literature. Int. J. Hyg. Environ. Heal..

[16] Niu J.F., Li Y., Wang W.L. (2013). Light-source-dependent role of nitrate and humic acid in tetracycline photolysis: Kinetics and mechanism. Chemosphere.

[17] Martinovic D., Hogarth W.T., Jones R.E., Sorensen P.W. (2007). Environmental estrogens suppress hormones, behavior, and reproductive fitness in male fathead minnows. Environ. Toxicol. Chem..

[18] Quinn B., Gagne F., Blaise C. (2008). An investigation into the acute and chronic toxicity of eleven pharmaceuticals (and their solvents) found in wastewater effluent on the cnidarian, hydra attenuata. Sci. Total Environ..

[19] Liu F., Ying G.G., Tao R., Zhao J.-L., Yang J.F., Zhao L.F. (2009). Effects of six selected antibiotics on plant growth and soil microbial and enzymatic activities. Environ. Pollut..

[20] Flint S., Markle T., Thompson S., Wallace E. (2012). Bisphenol a exposure, effects, and policy: A wildlife perspective. J. Environ. Manag..

[21] Kemper N. (2008). Veterinary antibiotics in the aquatic and terrestrial environment. Ecol. Indic..

[22] Hernando M.D., Mezcua M., Fernandez-Alba A.R., Barcelo D. (2006). Environmental risk assessment of pharmaceutical residues in wastewater effluents, surface waters and sediments. Talanta.

[23] Tacconelli E., de Angelis G., Cataldo M.A., Mantengoli E., Spanu T., Pan A., Corti G., Radice A., Stolzuoli L., Antinori S. (2009). Antibiotic usage and risk of colonization and infection with antibiotic-resistant bacteria: A hospital population-based study. Antimicrob. Agents Chemother..

[24] Kummerer K. (2009). Antibiotics in the aquatic environment—A review—Part II. Chemosphere.

[25] Baquero F., Martinez J.L., Canton R. (2008). Antibiotics and antibiotic resistance in water environments. Curr. Opin. Biotech..

[26] Behera S.K., Kim H.W., Oh J.-E., Park H.-S. (2011). Occurrence and removal of antibiotics, hormones and several other pharmaceuticals in wastewater treatment plants of the largest industrial city of Korea. Sci. Total Environ..

[27] Clara M., Strenn B., Gans O., Martinez E., Kreuzinger N., Kroiss H. (2005). Removal of selected pharmaceuticals, fragrances and endocrine disrupting compounds in a membrane bioreactor and conventional wastewater treatment plants. Water Res..

[28] Gros M., Petrovic M., Barcelo D. (2009). Tracing pharmaceutical residues of different therapeutic classes in environmental waters by using liquid chromatography/quadrupole-linear ion trap mass spectrometry and automated library searching. Anal. Chem..

[29] Miege C., Choubert J.M., Ribeiro L., Eusebe M., Coquery M. (2009). Fate of pharmaceuticals and personal care products in wastewater treatment plants—Conception of a database and first results. Environ. Pollut..

[30] Pailler J.Y., Krein A., Pfister L., Hoffmann L., Guignard C. (2009). Solid phase extraction coupled to liquid chromatography-tandem mass spectrometry analysis of sulfonamides, tetracyclines, analgesics and hormones in surface water and wastewater in luxembourg. Sci. Total Environ..

[31] Quintana J.B., Weiss S., Reemtsma T. (2005). Pathway's and metabolites of microbial degradation of selected acidic pharmaceutical and their occurrence in municipal wastewater treated by a membrane bioreactor. Water Res..

[32] Suarez S., Lema J.M., Omil F. (2010). Removal of pharmaceutical and personal care products (ppcps) under nitrifying and denitrifying conditions. Water Res..

[33] Thomas K.V., Dye C., Schlabach M., Langford K.H. (2007). Source to sink tracking of selected human pharmaceuticals from two oslo city hospitals and a wastewater treatment works. J. Environ. Monitor.

[34] Verlicchi P., Al Aukidy M., Galletti A., Petrovic M., Barcelo D. (2012). Hospital effluent: Investigation of the concentrations and distribution of pharmaceuticals and environmental risk assessment. Sci. Total Environ..

[35] Yu J.T., Bouwer E.J., Coelhan M. (2006). Occurrence and biodegradability studies of selected pharmaceuticals and personal care products in sewage effluent. Agr. Water Manag..

[36] Zorita S., Mårtensson L., Mathiasson L. (2009). Occurrence and removal of pharmaceuticals in a municipal sewage treatment system in the south of Sweden. Sci. Total Environ..

[37] Cardoso O., Porcher J.-M., Sanchez W. (2014). Factory-discharged pharmaceuticals could be a relevant source of aquatic environment contamination: Review of evidence and need for knowledge. Chemosphere.

[38] Boxall A.B.A. (2004). The environmental side effects of medication—How are human and veterinary medicines in soils and water bodies affecting human and environmental health?. EMBO Rep..

[39] Deo R.P. (2014). Pharmaceuticals in the surface water of the USA: A review. Curr. Environ. Health Rep..

[40] Gros M., Rodríguez-Mozaz S., Barceló D. (2012). Fast and comprehensive multi-residue analysis of a broad range of human and veterinary pharmaceuticals and some of their metabolites in surface and treated waters by ultra-high-performance liquid chromatography coupled to quadrupole-linear ion trap tandem mass spectrometry. J. Chromatogr. A.

[41] Vazquez-Roig P., Andreu V., Onghena M., Blasco C., Pico Y. (2011). Assessment of the occurrence and distribution of pharmaceuticals in a Mediterranean wetland (L'Albufera, Valencia, Spain) by LC-MS/MS. Anal. Bioanal. Chem..

[42] Vieno N.M., Harkki H., Tuhkanen T., Kronberg L. (2007). Occurrence of pharmaceuticals in river water and their elimination a pilot-scale drinking water treatment plant. Environ. Sci. Technol..

[43] Gracia-Lor E., Sancho J.V., Serrano R., Hernández F. (2012). Occurrence and removal of pharmaceuticals in wastewater treatment plants at the spanish mediterranean area of valencia. Chemosphere.

[44] Gulkowska A., Leung H.W., So M.K., Taniyasu S., Yamashita N., Yeunq L.W.Y., Richardson B.J., Lei A.P., Giesy J.P., Lam P.K.S. (2008). Removal of antibiotics from wastewater by sewage treatment facilities in hong kong and shenzhen, china. Water Res..

[45] Schlusener M.P., Bester K. (2005). Determination of steroid hormones, hormone conjugates and macrolide antibiotics in influents and effluents of sewage treatment plants utilising high-performance liquid chromatography/tandem mass spectrometry with electrospray and atmospheric pressure chemical ionisation. Rapid Commun. Mass Spectrom..

[46] Senta I., Terzic S., Ahel M. (2008). Simultaneous determination of sulfonamides, fluoroquinolones, macrolides and trimethoprim in wastewater and river water by LC-tandem-MS. Chromatographia.

[47] Christian T., Schneider R.J., Farber H.A., Skutlarek D., Meyer M.T., Goldbach H.E. (2003). Determination of antibiotic residues in manure, soil, and surface waters. Acta Hydroch. Hydrob..

[48] Luo Y., Xu L., Rysz M., Wang Y.Q., Zhang H., Alvarez P.J.J. (2011). Occurrence and transport of tetracycline, sulfonamide, quinolone, and macrolide antibiotics in the haihe river basin, China. Environ. Sci. Technol..

[49] Massey L.B., Haggard B.E., Galloway J.M., Loftin K.A., Meyer M.T., Green W.R. (2010). Antibiotic fate and transport in three effluent-dominated ozark streams. Ecol. Eng..

[50] Baran W., Adamek E., Ziemianska J., Sobczak A. (2011). Effects of the presence of sulfonamides in the environment and their influence on human health. J. Hazard. Mater..

[51] Batt A.L., Aga D.S. (2005). Simultaneous analysis of multiple classes of antibiotics by ion trap LC/MS/MS for assessing surface water and groundwater contamination. Anal. Chem..

[52] Batt A.L., Bruce I.B., Aga D.S. (2006). Evaluating the vulnerability of surface waters to antibiotic contamination from varying wastewater treatment plant discharges. Environ. Pollut..

[53] Batt A.L., Kim S., Aga D.S. (2007). Comparison of the occurrence of antibiotics in four full-scale wastewater treatment plants with varying designs and operations. Chemosphere.

[54] Botitsi E., Frosyni C., Tsipi D. (2007). Determination of pharmaceuticals from different therapeutic classes in wastewaters by liquid chromatography-electrospray ionization-tandem mass spectrometry. Anal. Bioanal. Chem..

[55] Gros M., Rodriguez-Mozaz S., Barcelo D. (2013). Rapid analysis of multiclass antibiotic residues and some of their metabolites in hospital, urban wastewater and river water by ultra-high-performance liquid chromatography coupled to quadrupole-linear ion trap tandem mass spectrometry. J. Chromatogr. A.

[56] Yang S.W., Carlson K. (2003). Evolution of antibiotic occurrence in a river through pristine, urban and agricultural landscapes. Water Res..

[57] Diaz-Cruz M.S., Garcia-Galan M.J., Barcelo D. (2008). Highly sensitive simultaneous determination of sulfonamide antibiotics and one metabolite in environmental waters by liquid chromatography-quadrupole linear ion trap-mass spectrometry. J. Chromatogr. A.

[58] García-Galán M.J., Díaz-Cruz M.S., Barceló D. (2011). Occurrence of sulfonamide residues along the ebro river basin: Removal in wastewater treatment plants and environmental impact assessment. Environ. Int..

[59] Karthikeyan K.G., Meyer M.T. (2006). Occurrence of antibiotics in wastewater treatment facilities in wisconsin, USA. Sci. Total Environ..

[60] Kim S., Eichhorn P., Jensen J.N., Weber A.S., Aga D.S. (2005). Removal of antibiotics in wastewater: Effect of hydraulic and solid retention times on the fate of tetracycline in the activated sludge process. Environ. Sci. Technol..

[61] Pena A., Paulo M., Silva L.J.G., Seifrtova M., Lino C.M., Solich P. (2010). Tetracycline antibiotics in hospital and municipal wastewaters: A pilot study in portugal. Anal. Bioanal. Chem..

[62] Vieno N.M., Tuhkanen T., Kronberg L. (2006). Analysis of neutral and basic pharmaceuticals in sewage treatment plants and in recipient rivers using solid phase extraction and liquid chromatography-tandem mass spectrometry detection. J. Chromatogr. A.

[63] Liu Z.H., Kanjo Y., Mizutani S. (2009). Removal mechanisms for endocrine disrupting compounds (edcs) in wastewater treatment—Physical means, biodegradation, and chemical advanced oxidation: A review. Sci. Total Environ..

[64] Manickum T., John W. (2014). Occurrence, fate and environmental risk assessment of endocrine disrupting compounds at the wastewater treatment works in Pietermaritzburg (South africa). Sci. Total Environ..

[65] Gorga M., Petrovic M., Barcelo D. (2013). Multi-residue analytical method for the determination of endocrine disruptors and related compounds in river and waste water using dual column liquid chromatography switching system coupled to mass spectrometry. J. Chromatogr. A.

[66] Silva C.P., Otero M., Esteves V. (2012). Processes for the elimination of estrogenic steroid hormones from water: A review. Environ. Pollut..

[67] Homem V., Santos L. (2011). Degradation and removal methods of antibiotics from aqueous matrices—A review. J. Environ. Manag..

[68] Onesios K.M., Bouwer E.J. (2012). Biological removal of pharmaceuticals and personal care products during laboratory soil aquifer treatment simulation with different primary substrate concentrations. Water Res..

[69] Muller M., Combalbert S., Delgenes N., Bergheaud V., Rocher V., Benoit P., Delgenes J.P., Patureau D., Hernandez-Raquet G. (2010). Occurrence of estrogens in sewage sludge and their fate during plant-scale anaerobic digestion. Chemosphere.

[70] Carballa M., Omil F., Ternes T., Lema J.M. (2007). Fate of pharmaceutical and personal care products (ppcps) during anaerobic digestion of sewage sludge. Water Res..

[71] Czajka C.P., Londry K.L. (2006). Anaerobic biotransformation of estrogens. Sci. Total Environ..

[72] Andersen H., Siegrist H., Halling-Sorensen B., Ternes T.A. (2003). Fate of estrogens in a municipal sewage treatment plant. Environ. Sci. Technol..

[73] Ren Y.X., Nakano K., Nomura M., Chiba N., Nishimura O. (2007). Effects of bacterial activity on estrogen removal in nitrifying activated sludge. Water Res..

[74] Servos M.R., Bennie D.T., Burnison B.K., Jurkovic A., McInnis R., Neheli T., Schnell A., Seto P., Smyth S.A., Ternes T.A. (2005). Distribution of estrogens, 17 beta-estradiol and estrone, in canadian municipal wastewater treatment plants. Sci. Total Environ..

[75] De Graaff M.S., Vieno N.M., Kujawa-Roeleveld K., Zeeman G., Temmink H., Buisman C.J.N. (2011). Fate of hormones and pharmaceuticals during combined anaerobic treatment and nitrogen removal by partial nitritation-anammox in vacuum collected black water. Water Res..

[76] Joss A., Keller E., Alder A.C., Göbel A., McArdell C.S., Ternes T., Siegrist H. (2005). Removal of pharmaceuticals and fragrances in biological wastewater treatment. Water Res..

[77] Jelic A., Gros M., Ginebreda A., Cespedes-Sanchez R., Ventura F., Petrovic M., Barcelo D. (2011). Occurrence, partition and removal of pharmaceuticals in sewage water and sludge during wastewater treatment. Water Res..

[78] Gao P., Ding Y.J., Li H., Xagoraraki I. (2012). Occurrence of pharmaceuticals in a municipal wastewater treatment plant: Mass balance and removal processes. Chemosphere.

[79] Gros M., Petrović M., Ginebreda A., Barceló D. (2010). Removal of pharmaceuticals during wastewater treatment and environmental risk assessment using hazard indexes. Environ. Int..

[80] Schlüsener M.P., Bester K. (2008). Behavior of steroid hormones and conjugates during wastewater treatment—A comparison of three sewage treatment plants. Clean-Soil Air Water.

[81] Martinez F., Lopez-Munoz M.J., Aguado J., Melero J.A., Arsuaga J., Sotto A., Molina R., Segura Y., Pariente M.I., Revilla A. (2013). Coupling membrane separation and photocatalytic oxidation processes for the degradation of pharmaceutical pollutants. Water Res..

[82] Margot J., Kienle C., Magnet A., Weil M., Rossi L., de Alencastro L.F., Abegglen C., Thonney D., Chevre N., Scharer M. (2013). Treatment of micropollutants in municipal wastewater: Ozone or powdered activated carbon?. Sci. Total Environ..

[83] Fernandez R.L., McDonald J.A., Khan S.J., Le-Clech P. (2014). Removal of pharmaceuticals and endocrine disrupting chemicals by a submerged membrane photocatalysis reactor (mpr). Sep. Purif. Technol..

[84] Miralles-Cuevas S., Audino F., Oller I., Sanchez-Moreno R., Perez J.A.S., Malato S. (2014). Pharmaceuticals removal from natural water by nanofiltration combined with advanced tertiary treatments (solar photo-fenton, photo-fenton-like fe(iii)-edds complex and ozonation). Sep. Purif. Technol..

[85] Andersson D.I., Hughes D. (2014). Microbiological effects of sublethal levels of antibiotics. Nat. Rev. Microbiol..

[86] Pollice A., Laera G., Saturno D., Giordano C. (2008). Effects of sludge retention time on the performance of a membrane bioreactor treating municipal sewage. J. Membrane Sci..

[87] Kaya Y., Ersan G., Vergili I., Gonder Z.B., Yilmaz G., Dizge N., Aydiner C. (2013). The treatment of pharmaceutical wastewater using in a submerged membrane bioreactor under different sludge retention times. J. Membrane Sci..

[88] Clara M., Kreuzinger N., Strenn B., Gans O., Kroiss H. (2005). The solids retention time—A suitable design parameter to evaluate the capacity of wastewater treatment plants to remove micropollutants. Water Res..

[89] Bernhard M., Muller J., Knepper T.R. (2006). Biodegradation of persistent polar pollutants in wastewater: Comparison of an optimised lab-scale membrane bioreactor and activated sludge treatment. Water Res..

[90] Kimura K., Hara H., Watanabe Y. (2005). Removal of pharmaceutical compounds by submerged membrane bioreactors (mbrs). Desalination.

[91] Cases V., Alonso V., Argandona V., Rodriguez M., Prats D. (2011). Endocrine disrupting compounds: A comparison of removal between conventional activated sludge and membrane bioreactors. Desalination.

[92] Sipma J., Osuna B., Collado N., Monclus H., Ferrero G., Comas J., Rodriguez-Roda I. (2010). Comparison of removal of pharmaceuticals in mbr and activated sludge systems. Desalination.

[93] Clouzot L., Doumenq P., Vanloot P., Roche N., Marrot B. (2010). Membrane bioreactors for 17 alpha-ethinylestradiol removal. J. Membrane Sci..

[94] Radjenovic J., Petrovic M., Barcelo D. (2007). Analysis of pharmaceuticals in wastewater and removal using a membrane bioreactor. Anal. Bioanal. Chem..

[95] Radjenovic J., Petrovic M., Barcelo D. (2009). Fate and distribution of pharmaceuticals in wastewater and sewage sludge of the conventional activated sludge (cas) and advanced membrane bioreactor (mbr) treatment. Water Res..

[96] Fan H., Li J., Zhang L., Feng L. (2014). Contribution of sludge adsorption and biodegradation to the removal of five pharmaceuticals in a submerged membrane bioreactor. Biochem. Eng. J..

[97] Nguyen L.N., Hai F.I., Kang J.G., Price W.E., Nghiem L.D. (2012). Removal of trace organic contaminants by a membrane bioreactor-granular activated carbon (mbr-gac) system. Bioresour. Technol..

[98] Nguyen L.N., Hai F.I., Yang S.F., Kang J.G., Leusch F.D.L., Roddick F., Price W.E., Nghiem L.D. (2013). Removal of trace organic contaminants by an mbr comprising a mixed culture of bacteria and white-rot fungi. Bioresour. Technol..

[99] Remy M., van der Marel P., Zwijnenburg A., Rulkens W., Temmink H. (2009). Low dose powdered activated carbon addition at high sludge retention times to reduce fouling in membrane bioreactors. Water Res..

[100] Chen Z.-B., He Z.-W., Tang C.-C., Hu D.-X., Cui Y.-B., Wang A.-J., Zhang Y., Yan L.-L., Ren N.-Q. (2014). Performance and model of a novel multi-sparger multi-stage airlift loop membrane bioreactor to treat high-strength 7-aca pharmaceutical wastewater: Effect of hydraulic retention time, temperature and ph. Bioresour. Technol..

[101] Dutta K., Lee M.-Y., Lai W.W.-P., Lee C.H., Lin A.Y.-C., Lin C.-F., Lin J.-G. (2014). Removal of pharmaceuticals and organic matter from municipal wastewater using two-stage anaerobic fluidized membrane bioreactor. Bioresour. Technol..

[102] Wright G.D. (2005). Bacterial resistance to antibiotics: Enzymatic degradation and modification. Adv. Drug Deliver. Rev..

[103] Fan C.A., He J.Z. (2011). Proliferation of antibiotic resistance genes in microbial consortia of sequencing batch reactors (sbrs) upon exposure to trace erythromycin or erythromycin-H2O. Water Res..

[104] Kim Y.H., Cha C.J., Cerniglia C.E. (2002). Purification and characterization of an erythromycin esterase from an erythromycin-resistant pseudomonas sp.. FEMS Microbiol. Lett..

[105] Baldrian P. (2006). Fungal laccases—Occurrence and properties. FEMS Microbiol. Rev..

[106] Demarche P., Junghanns C., Nair R.R., Agathos S.N. (2012). Harnessing the power of enzymes for environmental stewardship. Biotechnol. Adv..

[107] Durán N., Esposito E. (2000). Potential applications of oxidative enzymes and phenoloxidase-like compounds in wastewater and soil treatment: A review. Appl. Catal. B Environ..

[108] Aitken M.D. (1993). Waste treatment applications of enzymes: Opportunities and obstacles. Chem. Eng. J..

[109] Karam J., Nicell J.A. (1997). Potential applications of enzymes in waste treatment. J. Chem. Technol. Biot..

[110] Nyanhongo G.S., Gubitz G., Sukyai P., Leitner C., Haltrich D., Ludwig R. (2007). Oxidoreductases from trametes spp. In biotechnology: A wealth of catalytic activity. Food Technol. Biotech..

[111] De Gunzburg J., Bensoussan C. (2012). Methods for the Inactivation of Antibiotics.

[112] Cajthaml T., Kresinova Z., Svobodova K., Moder M. (2009). Biodegradation of endocrine-disrupting compounds and suppression of estrogenic activity by ligninolytic fungi. Chemosphere.

[113] Auriol M., Filali-Meknassi Y., Adams C.D., Tyagi R.D. (2006). Natural and synthetic hormone removal using the horseradish peroxidase enzyme: Temperature and ph effects. Water Res..

[114] Suzuki K., Hirai H., Murata H., Nishida T. (2003). Removal of estrogenic activities of 17 beta-estradiol and ethinylestradiol by ligninolytic enzymes from white rot fungi. Water Res..

[115] Tamagawa Y., Yamaki R., Hirai H., Kawai S., Nishida T. (2006). Removal of estrogenic activity of natural steroidal hormone estrone by ligninolytic enzymes from white rot fungi. Chemosphere.

[116] Auriol M., Filali-Meknassi Y., Tyagi R.D., Adams C.D. (2007). Oxidation of natural and synthetic hormones by the horseradish peroxidase enzyme in wastewater. Chemosphere.

[117] Melo C.F., Dezotti M. (2013). Evaluation of a horseradish peroxidase-catalyzed process for triclosan removal and antibacterial activity reduction. J. Chem. Technol. Biot..

[118] Zhang Y.J., Geissen S.U. (2010). *In vitro* degradation of carbamazepine and diclofenac by crude lignin peroxidase. J. Hazard. Mater..

[119] Wen X., Jia Y., Li J. (2009). Degradation of tetracycline and oxytetracycline by crude lignin peroxidase prepared from phanerochaete chrysosporium—A white rot fungus. Chemosphere.

[120] Wen X., Jia Y., Li J. (2010). Enzymatic degradation of tetracycline and oxytetracycline by crude manganese peroxidase prepared from phanerochaete chrysosporium. J. Hazard. Mater..

[121] Suda T., Hata T., Kawai S., Okamura H., Nishida T. (2012). Treatment of tetracycline antibiotics by laccase in the presence of 1-hydroxybenzotriazole. Bioresour. Technol..

[122] Hata T., Shintate H., Kawai S., Okamura H., Nishida T. (2010). Elimination of carbamazepine by repeated treatment with laccase in the presence of 1-hydroxybenzotriazole. J. Hazard. Mater..

[123] Giardina P., Faraco V., Pezzella C., Piscitelli A., Vanhulle S., Sannia G. (2010). Laccases: A never-ending story. Cell. Mol. Life Sci..

[124] Majeau J.-A., Brar S.K., Tyagi R.D. (2010). Laccases for removal of recalcitrant and emerging pollutants. Bioresour. Technol..

[125] Desai S.S., Nityanand C. (2011). Microbial laccases and their applications: A review. J. Biotechnol..

[126] Kudanga T., Nyanhongo G.S., Guebitz G.M., Burton S. (2011). Potential applications of laccase-mediated coupling and grafting reactions: A review. Enzyme Microb Tech..

[127] Rodríguez-Couto S., Toca Herrera J.L. (2006). Industrial and biotechnological applications of laccases: A review. Biotechnol. Adv..

[128] Chea V., Paolucci-Jeanjean D., Belleville M.P., Sanchez J. (2012). Optimization and characterization of an enzymatic membrane for the degradation of phenolic compounds. Catal. Today.

[129] Widsten P., Kandelbauer A. (2008). Laccase applications in the forest products industry: A review. Enzyme Microb. Tech..

[130] Auriol M., Filali-Meknassi Y., Tyagi R.D., Adams C.D. (2007). Laccase-catalyzed conversion of natural and synthetic hormones from a municipal wastewater. Water Res..

[131] Lloret L., Eibes G., Lú-Chau T.A., Moreira M.T., Feijoo G., Lema J.M. (2010). Laccase-catalyzed degradation of anti-inflammatories and estrogens. Biochem. Eng. J..

[132] Margot J., Maillard J., Rossi L., Barry D.A., Holliger C. (2013). Influence of treatment conditions on the oxidation of micropollutants by trametes versicolor laccase. New Biotechnol..

[133] Auriol M., Filali-Meknassi Y., Adams C.D., Tyagi R.D., Noguerol T.N., Pina B. (2008). Removal of estrogenic activity of natural and synthetic hormones from a municipal wastewater: Efficiency of horseradish peroxidase and laccase from trametes versicolor. Chemosphere.

[134] Weng S.-S., Ku K.-L., Lai H.-T. (2012). The implication of mediators for enhancement of laccase oxidation of sulfonamide antibiotics. Bioresour. Technol..

[135] Galli C., Gentili P. (2004). Chemical messengers: Mediated oxidations with the enzyme laccase. J. Phys. Org. Chem..

[136] Canas A.I., Camarero S. (2010). Laccases and their natural mediators: Biotechnological tools for sustainable eco-friendly processes. Biotechnol. Adv..

[137] Kurniawati S., Nicell J.A. (2007). Efficacy of mediators for enhancing the laccase-catalyzed oxidation of aqueous phenol. Enzyme Microb. Tech..

[138] Husain M., Husain Q. (2008). Applications of redox mediators in the treatment of organic pollutants by using oxidoreductive enzymes: A review. Crit. Rev. Environ. Sci. Technol..

[139] Banci L., Ciofi-Baffoni S., Tien M. (1999). Lignin and mn peroxidase-catalyzed oxidation of phenolic lignin oligomers. Biochemistry.

[140] Garcia H.A., Hoffman C.M., Kinney K.A., Lawler D.F. (2011). Laccase-catalyzed oxidation of oxybenzone in municipal wastewater primary effluent. Water Res..

[141] Inoue Y., Hata T., Kawai S., Okamura H., Nishida T. (2010). Elimination and detoxification of triclosan by manganese peroxidase from white rot fungus. J. Hazard. Mater..

[142] Catapane M., Nicolucci C., Menale C., Mita L., Rossi S., Mita D.G., Diano N. (2013). Enzymatic removal of estrogenic activity of nonylphenol and octylphenol aqueous solutions by immobilized laccase from trametes versicolor. J. Hazard. Mater..

[143] Nicolucci C., Rossi S., Menale C., Godjevargova T., Ivanov Y., Bianco M., Mita L., Bencivenga U., Mita D.G., Diano N. (2011). Biodegradation of bisphenols with immobilized laccase or tyrosinase on polyacrylonitrile beads. Biodegradation.

[144] Mateo C., Palomo J.M., Fernandez-Lorente G., Guisan J.M., Fernandez-Lafuente R. (2007). Improvement of enzyme activity, stability and selectivity via immobilization techniques. Enzyme Microb. Tech..

[145] Fernandez-Fernandez M., Sanroman M.A., Moldes D. (2013). Recent developments and applications of immobilized laccase. Biotechnol. Adv..

[146] Duran N., Rosa M.A., D'Annibale A., Gianfreda L. (2002). Applications of laccases and tyrosinases (phenoloxidases) immobilized on different supports: A review. Enzyme Microb. Tech..

[147] Brady D., Jordaan J. (2009). Advances in enzyme immobilisation. Biotechnol. Lett..

[148] Jochems P., Satyawali Y., Diels L., Dejonghe W. (2011). Enzyme immobilization on/in polymeric membranes: Status, challenges and perspectives in biocatalytic membrane reactors (bmrs). Green Chem..

[149] Hanefeld U., Gardossi L., Magner E. (2009). Understanding enzyme immobilisation. Chem. Soc. Rev..

[150] Sheldon R.A. (2007). Enzyme immobilization: The quest for optimum performance. Adv. Synth. Catal..

[151] Mori M., Garcia R.G., Belleville M.P., Paolucci-Jeanjean D., Sanchez J., Lozano P., Vaultier M., Rios G. (2005). A new way to conduct enzymatic synthesis in an active membrane using ionic liquids as catalyst support. Catal. Today.

[152] Cao L.Q. (2005). Immobilised enzymes: Science or art?. Curr. Opin. Chem. Biol..

[153] Cowan D.A., Fernandez-Lafuente R. (2011). Enhancing the functional properties of thermophilic enzymes by chemical modification and immobilization. Enzyme Microb. Tech..

[154] Plagemann R., Jonas L., Kragl U. (2011). Ceramic honeycomb as support for covalent immobilization of laccase from trametes versicolor and transformation of nuclear fast red. Appl. Microbiol. Biot..

[155] Dai Y.R., Niu J.F., Yin L.F., Xu J.J., Xu J.R. (2013). Laccase-carrying electrospun fibrous membrane for the removal of polycyclic aromatic hydrocarbons from contaminated water. Sep. Purif. Technol..

[156] Xu R., Zhou Q.J., Li F.T., Zhang B.R. (2013). Laccase immobilization on chitosan/poly(vinyl alcohol) composite nanofibrous membranes for 2,4-dichlorophenol removal. Chem. Eng. J..

[157] Lloret L., Hollmann F., Eibes G., Feijoo G., Moreira M.T., Lema J.M. (2012). Immobilisation of laccase on eupergit supports and its application for the removal of endocrine disrupting chemicals in a packed-bed reactor. Biodegradation.

[158] Cabana H., Alexandre C., Agathos S.N., Jones J.P. (2009). Immobilization of laccase from the white rot fungus coriolopsis polyzona and use of the immobilized biocatalyst for the continuous elimination of endocrine disrupting chemicals. Bioresour. Technol..

[159] Cabana H., Jones J.P., Agathos S.N. (2007). Preparation and characterization of cross-linked laccase aggregates and their application to the elimination of endocrine disrupting chemicals. J. Biotechnol..

[160] Lloret L., Eibes G., Feijoo G., Moreira M.T., Lema J.M. (2012). Continuous operation of a fluidized bed reactor for the removal of estrogens by immobilized laccase on eupergit supports. J. Biotechnol..

[161] Sanchez Marcano J.G., Tsotsis T.T. (2002). Catalytic Membranes and Membrane Reactors.

[162] Nguyen L.N., Hai F.I., Price W.E., Leusch F.D.L., Roddick F., Ngo H.H., Guo W., Magram S.F., Nghiem L.D. (2014). The effects of mediator and granular activated carbon addition on degradation of trace organic contaminants by an enzymatic membrane reactor. Bioresour. Technol..

[163] Paolucci-Jeanjean D., Belleville M.P., Rios G.M., Zakhia N. (2000). The effect of enzyme concentration and space time on the performance of a continuous recycle membrane reactor for one-step starch hydrolysis. Biochem. Eng. J..

[164] Grzeskowiak-Przywecka A., Slominska L. (2007). Saccharification of potato starch in an ultrafiltration reactor. J. Food Eng..

[165] Perea A., Ugalde U. (1996). Continuous hydrolysis of whey proteins in a membrane recycle reactor. Enzyme Microb. Tech..

[166] Cabrera-Padilla R.Y., Pinto G.A., Giordano R.L.C., Giordano R.C. (2009). A new conception of enzymatic membrane reactor for the production of whey hydrolysates with low contents of phenylalanine. Process. Biochem..

[167] Calabro V., Curcio S., de Paola M.G., Iorio G. (2009). Optimization of membrane bioreactor performances during enzymatic oxidation of waste bio-polyphenols. Desalination.

[168] Chhabra M., Mishra S., Sreekrishnan T.R. (2009). Laccase/mediator assisted degradation of triarylmethane dyes in a continuous membrane reactor. J. Biotechnol..

[169] Lloret L., Eibes G., Feijoo G., Moreira M.T., Lema J.M. (2012). Degradation of estrogens by laccase from myceliophthora thermophila in fed-batch and enzymatic membrane reactors. J. Hazard. Mater..

[170] Escalona I., de Grooth J., Font J., Nijmeijer K. (2014). Removal of bpa by enzyme polymerization using nf membranes. J. Membrane Sci..

[171] Nguyen L.N., Hai F.I., Price W.E., Leusch F.D.L., Roddick F., McAdam E.J., Magram S.F., Nghiem L.D. (2014). Continuous biotransformation of bisphenol a and diclofenac by laccase in an enzymatic membrane reactor. Int. Biodeter. Biodegr..

[172] Mendoza L., Jonstrup M., Hatti-Kaul R., Mattiasson B. (2011). Azo dye decolorization by a laccase/mediator system in a membrane reactor: Enzyme and mediator reusability. Enzyme Microb. Tech..

[173] Gasser C.A., Yu L., Svojitka J., Wintgens T., Ammann E.M., Shahgaldian P., Corvini P.F.X., Hommes G. (2014). Advanced enzymatic elimination of phenolic contaminants in wastewater: A nano approach at field scale. Appl. Microbiol. Biot..

[174] Rios G.M., Belleville M.P., Paolucci D., Sanchez J. (2004). Progress in enzymatic membrane reactors - a review. J. Membrane Sci..

[175] Belleville M.-P., Paolucci-Jeanjean D., Rios G.M. (2010). Separation, extraction and concentration processes in the food, beverage and nutraceutical industries (membrane bioreactors and the production of food ingredients). Woodead Food Ser..

[176] Kanwar L., Goswami P. (2002). Isolation of a pseudomonas lipase produced in pure hydrocarbon substrate and its application in the synthesis of isoamyl acetate using membrane-immobilised lipase. Enzyme Microb. Tech..

[177] Tan T.W., Wang F., ZHang H. (2002). Preparation of pva/chitosan lipase membrane reactor and its application in synthesis of monoglyceride. J. Mol. Catal. B Enzym..

[178] Hilal N., Nigmatullin R., Alpatova A. (2004). Immobilization of cross-linked lipase aggregates within microporous polymeric membranes. J. Membrane Sci..

[179] Torras C., Nabarlatz D., Vallot G., Montane D., Garcia-Valls R. (2008). Composite polymeric membranes for process intensification: Enzymatic hydrolysis of oligodextrans. Chem. Eng. J..

[180] Sousa H.A., Rodrigues C., Klein E., Afonso C.A.M., Crespo J.G. (2001). Immobilisation of pig liver esterase in hollow fibre membranes. Enzyme Microb. Tech..

[181] Xu J., Wang Y.J., Hu Y., Luo G.S., Dai Y.Y. (2006). Immobilization of lipase by filtration into a specially designed microstructure in the ca/ptfe composite membrane. J. Mol. Catal. B Enzym..

[182] Wang Y.J., Jian X., Luo G.S., Dai Y.Y. (2008). Immobilization of lipase by ultrafiltration and cross-linking onto the polysulfone membrane surface. Bioresour. Technol..

[183] Sakaki K., Giorno L., Drioli E. (2001). Lipase-catalyzed optical resolution of racemic naproxen in biphasic enzyme membrane reactors. J. Membrane Sci..

[184] Trusek-Holownia A., Noworyta A. (2007). An integrated process: Ester synthesis in an enzymatic membrane reactor and water sorption. J. Biotechnol..

[185] Shamel M.M., Ramachandran K.B., Hasan M., Al-Zuhair S. (2007). Hydrolysis of palm and olive oils by immobilised lipase using hollow fibre reactor. Biochem. Eng. J..

[186] Paiva A.L., Balcao V.M., Malcata F.X. (2000). Kinetics and mechanisms of reactions catalyzed by immobilized lipases. Enzyme Microb. Tech..

[187] Belleville M.P., Lozano P., Iborra J.L., Rios G.M. (2001). Preparation of hybrid membranes for enzymatic reaction. Sep. Purif. Technol..

[188] Gumi T., Paolucci-Jeanjean D., Belleville M.P., Rios G.A. (2007). Enzymatic membrane reactor involving a hybrid membrane in supercritical carbon dioxide. J. Membrane Sci..

[189] Lozano P., Perez-Marin A.B., de Diego T., Gomez D., Paolucci-Jeanjean D., Belleville M.P., Rios G.M., Iborra J.L. (2002). Active membranes coated with immobilized candida antarctica lipase b: Preparation and application for continuous butyl butyrate synthesis in organic media. J. Membrane Sci..

[190] Durante D., Casadio R., Martelli L., Tasco G., Portaccio M., de Luca P., Bencivenga U., Rossi S., Di Martino S., Grano V. (2004). Isothermal and non-isothermal bioreactors in the detoxification of waste waters polluted by aromatic compounds by means of immobilised laccase from rhus vernicifera. J. Mol. Catal. B Enzym..

[191] Lopez-Gallego F., Betancor L., Mateo C., Hidalgo A., Alonso-Morales N., Dellamora-Ortiz G., Guisan J.M., Fernandez-Lafuente R. (2005). Enzyme stabilization by glutaraldehyde crosslinking of adsorbed proteins on aminated supports. J. Biotechnol..

[192] Hou J., Dong G., Ye Y., Chen V. (2014). Laccase immobilization on titania nanoparticles and titania-functionalized membranes. J. Membrane Sci..

[193] Akay G., Erhan E., Keskinler B., Algur O.F. (2002). Removal of phenol from wastewater using membrane-immobilized enzymes—part II. Cross-flow filtration. J. Membrane Sci..

[194] Erhan E., Keskinler B., Akay G., Algur O.F. (2002). Removal of phenol from water by membrane-immobilized enzymes—Part I. Dead-end filtration. J. Membrane Sci..

[195] Diano N., Grano V., Fraconte L., Caputo P., Ricupito A., Attanasio A., Bianco M., Bencivenga U., Rossi S., Manco I. (2007). Non-isothermal bioreactors in enzymatic remediation of waters polluted by endocrine disruptors: Bpa as a model of pollutant. Appl. Catal. B Environ..

[196] Lozano P., de Diego T., Belleville M.P., Rios G.M., Iborra J.L. (2000). A dynamic membrane reactor with immobilized alpha-chymotrypsin for continuous kyotorphin synthesis in organic media. Biotechnol. Lett..

[197] De Cazes M., Belleville M.-P., Petit E., Llorca M., Rodríguez-Mozaz S., de Gunzburg J., Barceló D., Sanchez-Marcano J. (2014). Design and optimization of an enzymatic membrane reactor for tetracycline degradation. Catal. Today.

[198] Bohdziewicz J. (1998). Biodegradation of phenol by enzymes from pseudomonas sp. Immobilized onto ultrafiltration membranes. Process. Biochem..

[199] Lante A., Crapisi A., Krastanov A., Spettoli P. (2000). Biodegradation of phenols by laccase immobilised in a membrane reactor. Process. Biochem..

[200] Moeder M., Martin C., Koeller G. (2004). Degradation of hydroxylated compounds using laccase and horseradish peroxidase immobilized on microporous polypropylene hollow fiber membranes. J. Membrane Sci..

[201] Hou J., Dong G., Ye Y., Chen V. (2014). Enzymatic degradation of bisphenol-a with immobilized laccase on TiO_2_ sol–gel coated pvdf membrane. J. Membrane Sci..

[202] Burton S.G., Boshoff A., Edwards W., Rose P.D. (1998). Biotransformation of phenols using immobilised polyphenol oxidase. J. Mol. Catal. B Enzym..

[203] Edwards W., Bownes R., Leukes W.D., Jacobs E.P., Sanderson R., Rose P.D., Burton S.G. (1999). A capillary membrane bioreactor using immobilized polyphenol oxidase for the removal of phenols from industrial effluents. Enzyme Microb. Tech..

[204] Edwards W., Leukes W.D., Rose P.D., Burton S.G. (1999). Immobilization of polyphenol oxidase on chitosan-coated polysulphone capillary membranes for improved phenolic effluent bioremediation. Enzyme Microb. Tech..

[205] Boshoff A., Edwards W., Leukes W.D., Rose P.D., Burton S.G. (1998). Immobilisation of polyphenol oxidase on nylon and polyethersulphone membranes: Effect on product formation. Desalination.

[206] Vasileva N., Godjevargova T., Ivanova D., Gabrovska K. (2009). Application of immobilized horseradish peroxidase onto modified acrylonitrile copolymer membrane in removing of phenol from water. Int. J. Biol. Macromol..

[207] Jolivalt C., Brenon S., Caminade E., Mougin C., Pontie M. (2000). Immobilization of laccase from trametes versicolor on a modified pvdf microfiltration membrane: Characterization of the grafted support and application in removing a phenylurea pesticide in wastewater. J. Membrane Sci..

